# Exploring the causal role of multiple metabolites on ovarian cancer: a two sample Mendelian randomization study

**DOI:** 10.1186/s13048-023-01340-w

**Published:** 2024-01-23

**Authors:** Shaoxuan Liu, Danni Ding, Fangyuan Liu, Ying Guo, Liangzhen Xie, Feng-Juan Han

**Affiliations:** 1 First Clinical Medical College, Heilongjiang University of Chinese Medicine, Harbin, 150040 China; 2 Department of Obstetrics and Gynecology, The First Affiliated Hospital of Heilongjiang University of Chinese Medicine, Harbin, 150040 China

**Keywords:** Ovarian cancer, Causality, Genetically determined metabolites, Mendelian randomization

## Abstract

**Background:**

The mechanisms and risk factors underlying ovarian cancer (OC) remain under investigation, making the identification of new prognostic biomarkers and improved predictive factors critically important. Recently, circulating metabolites have shown potential in predicting survival outcomes and may be associated with the pathogenesis of OC. However, research into their genetic determinants is limited, and there are some inadequacies in understanding the distinct subtypes of OC. In this context, we conducted a Mendelian randomization study aiming to provide evidence for the relationship between genetically determined metabolites (GDMs) and the risk of OC and its subtypes.

**Methods:**

In this study, we consolidated genetic statistical data of GDMs with OC and its subtypes through a genome-wide association study (GWAS) and conducted a two-sample Mendelian randomization (MR) analysis. The inverse variance weighted (IVW) method served as the primary approach, with MR-Egger and weighted median methods employed for cross-validation to determine whether a causal relationship exists between the metabolites and OC risk. Moreover, a range of sensitivity analyses were conducted to validate the robustness of the results. MR-Egger intercept, and Cochran’s Q statistical analysis were used to evaluate possible heterogeneity and pleiotropy. False discovery rate (FDR) correction was applied to validate the findings. We also conducted a reverse MR analysis to validate whether the observed blood metabolite levels were influenced by OC risk. Additionally, metabolic pathway analysis was carried out using the MetaboAnalyst 5.0 software.

**Results:**

In MR analysis, we discovered 18 suggestive causal associations involving 14 known metabolites, 8 metabolites as potential risk factors, and 6 as potential cancer risk reducers. In addition, three significant pathways, "caffeine metabolism," "arginine biosynthesis," and "citrate cycle (TCA cycle)" were associated with the development of mucinous ovarian cancer (MOC). The pathways "caffeine metabolism" and "alpha-linolenic acid metabolism" were associated with the onset of endometrioid ovarian cancer (OCED).

**Conclusions:**

Our MR analysis revealed both protective and risk-associated metabolites, providing insights into the potential causal relationships between GDMs and the metabolic pathways related to OC and its subtypes. The metabolites that drive OC could be potential candidates for biomarkers.

**Supplementary Information:**

The online version contains supplementary material available at 10.1186/s13048-023-01340-w.

## Background

Ovarian cancer (OC) is the most challenging and daunting disease among all gynecological malignancies [1]. Due to its lack of typical early clinical symptoms and specific detection methods [2], patients often miss the optimal opportunity for chemotherapy and molecular targeted therapy. Furthermore, because of the gaps in the identification of prognostic biomarkers and targeted drugs for OC, the high recurrence rate and the emergence of drug resistance lead to a poor prognosis for OC patients [3].

Ovarian carcinogenesis is a complex multifactorial process, the possible causes include abnormal ovulatory cycles [4], chronic inflammation of the fallopian tubes [5], and gene mutations like Breast Cancer Gene 1 (BRCA1) [6]. Among these, metabolic dysregulation is considered one of the significant contributors [1, 7]. For instance, it is posited that local metabolic changes in the adipose tissue of obese individuals lead to various systemic metabolic alterations, such as insulin resistance, hyperglycemia, and chronic inflammation. These conditions more readily shape the tumor microenvironment, facilitating tumor initiation and progression [8]. In addition, cancer is fundamentally a disorder of cell growth and proliferation. During tumor initiation and development, cellular metabolism undergoes changes [9, 10], leading to meet the unrestrained proliferation energy needs of cancer cells and the synthesis of nucleic acids, proteins, and lipids. These metabolites act as cofactors or substrates, participating in enzymatic reactions involved in cancer cell epigenetic modifications and transcriptional regulation. Aberrant epigenetic regulatory modifications can further induce tumor development through metabolic reprogramming in cancer cells [11].

The molecular interaction network based on metabolomics offers fresh perspectives for elucidating the molecular mechanisms of OC treatment, discovering new therapeutic targets, and identifying reliable and effective biomarkers. Numerous metabolic groups and classes are associated with OC risk, including organic acids and their derivatives [12]. For example, studies have shown that circulating levels of pseudouridine in plasma are associated with a higher risk of developing OC 3-23 years prior to diagnosis [13]. Additionally, some scholars believe that the spectra of amino acids and organic acids can serve as potential screening tools for epithelial ovarian cancer (EOC) [14]. Currently, due to the following factors, these studies in OC remain less than satisfactory: (i) Intermediate metabolites have not been comprehensively studied. (ii) Most of the existing databases only contain distinct information about high-grade serous ovarian cancer (HGSOC) and lack histological types of other ovarian cancers. (iii) The absence of large-sample studies makes it difficult to explore the relationship between metabolites and OC in clinical practice [15].

Mendelian randomization (MR) serves as a powerful epidemiological tool that can effectively eliminate confounders and reveal potential causal relationships. Studies indicate that genetic polymorphisms affect biochemical levels in serum, suggesting that genetic variations might play a role in racial differences in the gender and/or age-related variations of circulating metabolite levels [16, 17]. A recent robust study on the GWAS of metabolites has pinpointed loci associated with the disease [16]. Moreover, developments by So-Youn Shin [17] on the database of genotype-dependent metabolic phenotypes, also known as genetically determined metabolites (GDM), have matched hundreds of metabolites and pathways with genetic data. This paves the way for further research into the potential relationship between serum metabolites in humans and associated genetic variations in the biological mechanisms of OC initiation and progression.

Our study aims to comprehensively investigate the causal relationship between various subtypes of OC and serum metabolic factors. Further, it provides reverse validation to ensure the directional accuracy of the results. By identifying metabolic pathways that may shed light on the mechanisms underlying the initiation of OC, this research offers practical and targeted guidance for the early detection, treatment, and prevention of high-risk OC patients and those with different OC subtypes.

## Materials and methods

### Study design

**Fig. 1:**
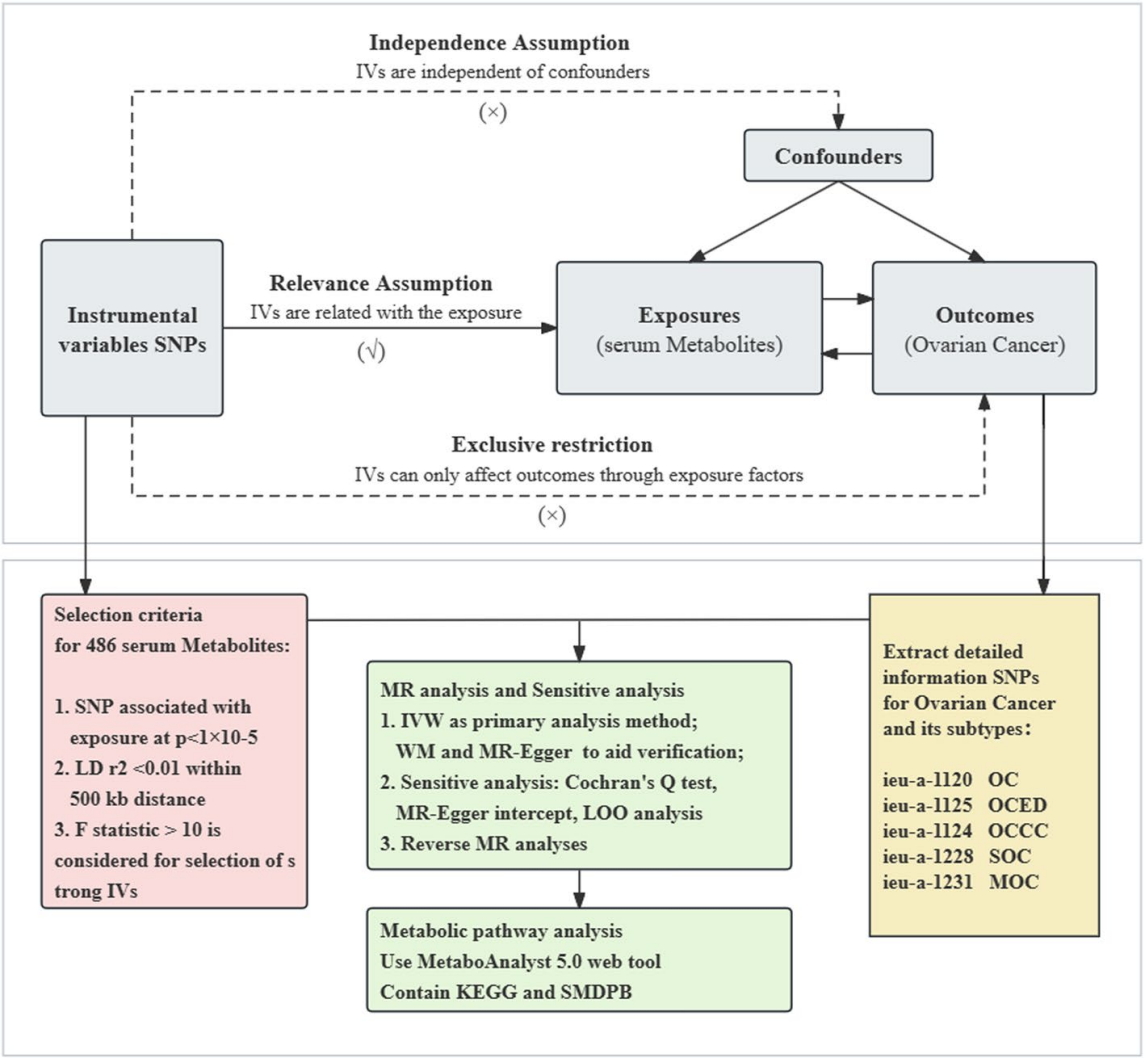
The flowchart of the study: the whole workflow of MR analysis. Abbreviations: *GWAS*, Genome-wide association studies; *SNPs*, Single nucleotide polymorphisms; *LOO*, Leave-One-Out; *OC*, Ovarian cancer; *OCED*, Endometrioid ovarian cancer; *OCCC*, Clear cell ovarian cancer; *SOC*, Serous ovarian cancer; *MOC*, Mucinous ovarian cancer; *MR*, Mendelian randomization; *IVW*, Inverse variance weighted; *WM*, weighted median

We systematically evaluated the causal relationship between 486 serum metabolites and OC risk using a MR design with two independent samples. Based on the STROBE-MR checklist [18] (Supplement file S1), a properly designed MR study relies on three basic assumptions: (i) genetically determined variations should exhibit a strong association with the exposure; (ii) genetically determined variations should be independent of confounding factors between the exposure and outcome; and (iii) genetically determined variations should only influence the outcome through the exposure and not via other pathways [19]. An overview of this study is illustrated in Fig. 1.

### Data sources

#### Data source for exposure

We obtained the genome-wide summary data involving 486 serum metabolites from the GWAS server of metabolomics (http://metabolomics.helmholtz-muenchen.de/gwas/). This dataset was generated by Shin et al. in 2014 through liquid chromatography and gas chromatography coupled with tandem mass spectrometry analysis of blood or plasma samples from 7,824 individuals of European ancestry [17]. It represents the most comprehensive report to date on genetic loci related to blood metabolites. A total of 529 metabolites were analyzed in the study, with strict quality control measures applied. Among these, 486 metabolites were available for genetic analysis, consisting of 309 known metabolites and 177 unknown metabolites. Furthermore, based on the Kyoto Encyclopedia of Genes and Genomes (KEGG) database [20], the 309 known metabolites were further classified into eight categories: cofactors and vitamins, energy, amino acids, carbohydrates, lipids, nucleotides, peptides, and xenobiotics (Supplementary Table S1).

#### Data source for outcome

The summarized data on OC included in this study was sourced from a genome-wide association study (GWAS) conducted by the Ovarian Cancer Association Consortium (OCAC). The GWAS included a total of 25,509 OC cases and 40,941 European ancestry controls [21]. To investigate the impact of serum metabolites on different types of OC, subgroup analyses were conducted using data specific to each OC subtype. An overview of the data relevant to OC can be obtained from the IEU Open GWAS project (https://gwas.mrcieu.ac.uk/) (Table 1).

**Table 1:** Gynecological cancers GWAS samples used in this study

**GWAS ID**	**Trait**	**No.Case**	**No.Control**	**Sample size**	**Year**	**Consortium**	**Populations**	**Reference**
ieu-a-1120	OC	25,509	40,941	66,450	2017	OCAC	European	Phelan, et al. [20]
ieu-a-1125	OCED	2,810	40,941	43,751				
ieu-a-1124	OCCC	1,366	40,941	42,307				
ieu-a-1228	SOC	14,049	40,941	54,990				
ieu-a-1231	MOC	2,566	40,941	43,507				

*Abbreviations*: *OC* Ovarian cancer, *OCED* Endometrioid ovarian cancer, *OCCC* Clear cell ovarian cancer, *SOC* Serous ovarian cancer, *MOC* Mucinous ovarian cancer

### Instrument variable selection

To ensure validity and precision of the findings associated with the relationship between GDMs regulators and risk of OC, the following quality control measures were implemented: (1) Given the non-independence of metabolites, the genome-wide significance threshold (*p*<5×10^-8^) might be overly conservative, possibly leading to the omission of potentially meaningful results [22] (Specific information can be found at Supplement Table S2). Consequently, we opted for a locus-specific significance threshold (*p* < 1 × 10^-5^, *r*^2^ < 0.1, 500kb), which has been widely employed in previous MR studies [23]. (2) The selected SNPs were matched within the dataset of the outcome (OC). For SNPs that could not be matched in the outcome dataset, we looked for proxies with an r^2^ threshold of >0.8, excluding those without any proxy (3). Finally, to quantitatively verify whether the selected SNPs were strong instruments, we calculated the F statistic and the proportion of variance explained (R^2^) for each instrument variable in relation to the exposure trait. Typically, F statistic > 10 was considered for selection of strong instrumental variables (IVs) [24]. (4) To maintain stability in our results, we retained only serum metabolites with a minimum of three instrument variables (5). To further ascertain the probability of detecting an effect and enhance the reliability of our study, we utilized an online power calculator (https://shiny.cnsgenomics.com/mRnd/) to compute the statistical power of our analyses.

### Statistical analysis

#### Mendelian randomization analysis

In our study, the inverse variance weighted (IVW) method was used as the primary approach to assess causal relationships between exposure and outcome. This method assumes the absence of horizontal pleiotropy across all IVs, under which the IVW method provides the most accurate causal estimation between exposure and outcome [25]. Additionally, we conducted several supplementary analyses to validate the robustness of our results. The MR-Egger method was employed to provide unbiased causal estimates in the presence of horizontal pleiotropy, and the intercept of this method was also used to detect horizontal pleiotropy [26]. When at least 50% of the IVs were valid, weighted median (WM) provided robust causal estimation [27]. Results were considered more robust if *P* < 0.05 for two or more MR methods [28]. Cochran's Q test was conducted to assess heterogeneity among the available SNPs [29]. To verify that the obtained causal estimates were not driven by individual SNPs, we performed a leave-one-out analysis by removing each SNP and examining if the previous causal relationship was altered [30]. Finally, scatter plots and funnel plots were used to visually display the relationships and interplay between each genetic instrument. FDR (false discovery rate) correction was applied to correct for multiple comparisons. A *P* < 0.05 before FDR correction was considered as suggestive for association. All MR analyses were conducted using the "TwoSampleMR" package (version 0.4.22). It is worth mentioning that the R package can use effect allele frequencies to automatically harmonize the exposure and outcome datasets, ensuring that the effect of the SNP on the exposure and the effect of the SNP on the outcome corresponding to the same allele.

#### Reverse Mendelian randomization

To investigate whether the outcomes studied had an impact on serum metabolite levels, we performed a reverse MR analysis. In this reverse analysis, we utilized SNPs selected from data on OC and its subtypes as IVs, with the chosen blood metabolite as the outcome, to explore whether the previously determined relationship was bidirectional.

#### Metabolic pathway analysis

Metabolic pathway analysis was conducted using the network-based MetaConflic 5.0, available at https://www.metaboanalyst.ca/ [31]. Two databases, namely the Small Molecule Pathway Database (SMPDB) and the Kyoto Encyclopedia of Genes and Genomes (KEGG) database, were utilized in this study. The significance level for pathway analysis was set at 0.05.

## Results

### Selection of instrumental variables

After undergoing a rigorous selection process, we conducted Mendelian random analysis on the relationship between 486 blood metabolites and OC and its subtypes. To ensure the robustness of our results, the study only retained metabolites that contained at least 3 SNPs, resulting in a total of 485 unique metabolites. The F-statistics for all SNPs involved were greater than 10, indicating that our results are less likely to be affected by weak IV bias. Specific information regarding IVs can be found in Supplementary Table S3. The total R^2^ and the median F-statistic (and range) for each metabolite in Supplementary Table S4.

### Causal estimation of blood metabolites on OC and its subtypes

**Fig. 2:**
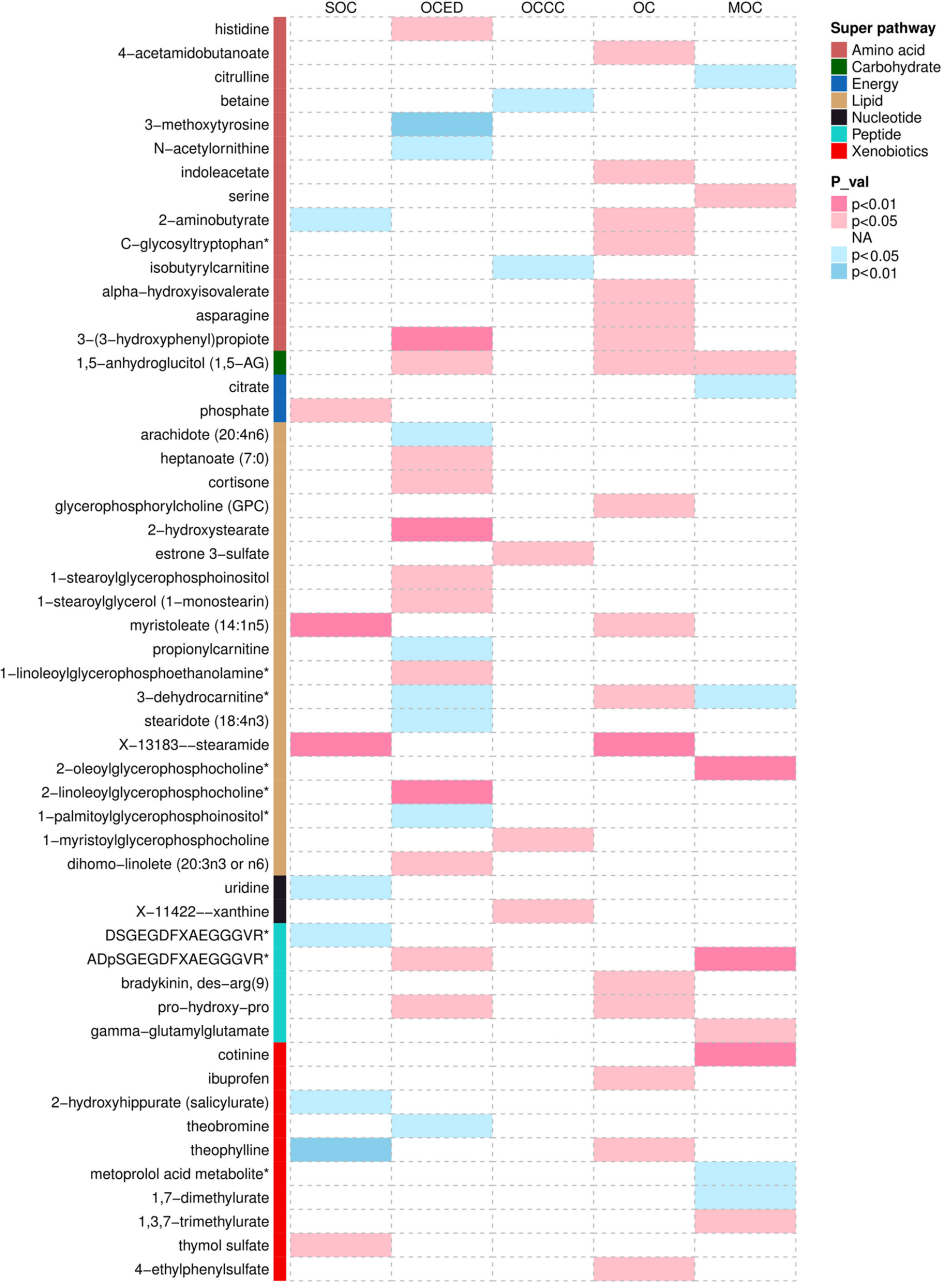
Mendelian randomization associations of known metabolites on the risk of the four different GWAS datasets for five OC phenotypes. The pink group represents risk factors, while the blue group represents protective factors. Abbreviations: *OC,* Ovarian cancer*; OCED,* Endometrioid ovarian cancer*; OCCC,* Clear cell ovarian cancer*; SOC,* Serous ovarian cancer*; MOC,* Mucinous ovarian cancer

**Fig. 3:**
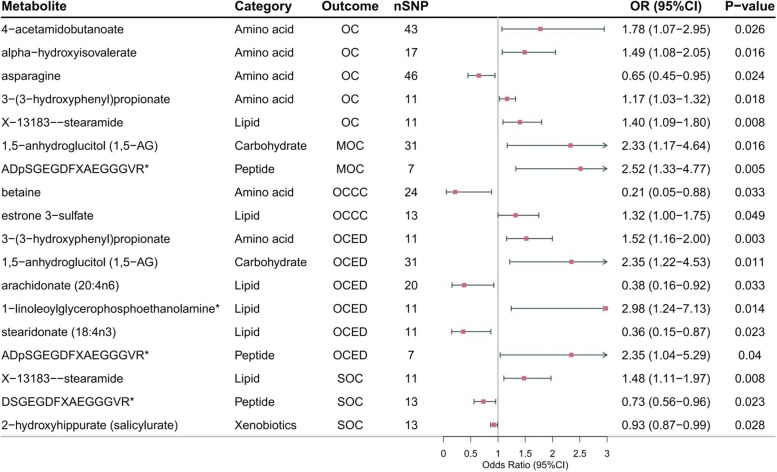
Forest plots illustrating the effect estimates for the identified candidate metabolite associations with several OC phenotypes based on the IVW MR model. Abbreviations: *SNPs*, Single nucleotide polymorphisms; *OC,* Ovarian cancer*; OCED,* Endometrioid ovarian cancer*; OCCC,* Clear cell ovarian cancer*; SOC,* Serous ovarian cancer*; MOC,* Mucinous ovarian cancer*; OR,* Odds Ratio

We conducted MR analysis on 486 blood metabolites and five subtypes of OC, and discovered 112 suggestive causal associations (IVW *P* < 0.05), involving 53 known metabolites as shown in Fig. 2. To ensure the robustness of our results, we further screened the 112 suggestive associations. We included metabolites with consistent causal associations identified by at least three MR methods, including the IVW, WM and MR-Egger methods. In total, we retained 18 causal associations involving 14 known metabolites, 8 metabolites as potential risk factors, and 6 as potential cancer risk reducers (Table 2 and Fig. 3).

**Table 2:** Mendelian randomization estimates for the identified candidate metabolite associations with OC phenotypes

Outcome	Exposure	Method	nsnp	*P*-value	OR	95%CI	P_FDR_
OC	4-acetamidobutanoate	IVW	43	0.026	1.78	1.07-2.95	0.772322416
		WM	43	0.024	2.13	1.10-4.11	
		MR Egger	43	0.008	6.45	1.76-23.72	
OC	alpha-hydroxyisovalerate	IVW	17	0.016	1.49	1.08-2.05	0.65956674
		WM	17	0.035	1.60	1.03-2.48	
		MR Egger	17	0.254	1.76	0.69-4.46	
OC	asparagine	IVW	46	0.024	0.65	0.45-0.95	0.772322416
		WM	46	0.004	0.44	0.25-0.77	
		MR Egger	46	0.028	0.42	0.19-0.89	
OC	3-(3-hydroxyphenyl) propionate	IVW	11	0.018	1.17	1.03-1.32	0.65956674
		WM	11	0.040	1.20	1.01-1.44	
		MR Egger	11	0.428	1.16	0.82-1.63	
OC	X-13183--stearamide	IVW	11	0.008	1.40	1.09-1.80	0.65956674
		WM	11	0.025	1.49	1.05-2.12	
		MR Egger	11	0.158	1.49	0.90-2.48	
MOC	1,5-anhydroglucitol (1,5-AG)	IVW	31	0.016	2.33	1.17-4.64	0.851594997
		WM	31	0.023	3.21	1.18-8.77	
		MR Egger	31	0.052	5.77	1.06-31.43	
MOC	ADpSGEGDFXAEGGGVR	IVW	7	0.005	2.52	1.33-4.77	0.851594997
		WM	7	0.003	3.48	1.54-7.89	
		MR Egger	7	0.232	3.93	0.55-28.20	
OCCC	betaine	IVW	24	0.033	0.21	0.05-0.88	0.981998335
		WM	24	0.013	0.10	0.02-0.61	
		MR Egger	24	0.105	0.05	0.00-1.59	
OCCC	estrone 3-sulfate	IVW	13	0.049	1.32	1.00-1.75	0.981998335
		WM	13	0.014	1.59	1.10-2.29	
		MR Egger	13	0.408	1.21	0.78-1.88	
OCED	3-(3-hydroxyphenyl) propionate	IVW	11	0.003	1.52	1.16-2.00	0.693730355
		WM	11	0.005	1.70	1.17-2.46	
		MR Egger	11	0.045	2.34	1.14-4.77	
OCED	1,5-anhydroglucitol (1,5-AG)	IVW	31	0.011	2.35	1.22-4.53	0.693730355
		WM	31	0.030	2.65	1.10-6.40	
		MR Egger	31	0.406	2.00	0.40-10.07	
OCED	arachidonate (20:4n6)	IVW	20	0.033	0.38	0.16-0.92	0.748839011
		WM	20	0.007	0.20	0.06-0.64	
		MR Egger	20	0.031	0.17	0.04-0.75	
OCED	1-linoleoylglycerophosphoethanolamine	IVW	11	0.014	2.98	1.24-7.13	0.693730355
		WM	11	0.025	4.18	1.19-14.66	
		MR Egger	11	0.181	5.56	0.55-56.35	
OCED	stearidonate (18:4n3)	IVW	11	0.023	0.36	0.15-0.87	0.694101869
		WM	11	0.039	0.30	0.09-0.94	
		MR Egger	11	0.098	0.10	0.01-1.15	
OCED	ADpSGEGDFXAEGGGVR	IVW	7	0.040	2.35	1.04-5.29	0.748839011
		WM	7	0.009	2.94	1.30-6.62	
		MR Egger	7	0.023	19.77	3.22-121.42	
SOC	X-13183--stearamide	IVW	11	0.008	1.48	1.11-1.97	0.871510468
		WM	11	0.026	1.58	1.06-2.37	
		MR Egger	11	0.150	1.61	0.89-2.90	
SOC	DSGEGDFXAEGGGVR	IVW	13	0.023	0.73	0.56-0.96	0.952113534
		WM	13	0.024	0.65	0.44-0.94	
		MR Egger	13	0.092	0.47	0.21-1.05	
SOC	2-hydroxyhippurate (salicylurate)	IVW	13	0.028	0.93	0.87-0.99	0.952113534
		WM	13	0.035	0.90	0.82-0.99	
		MR Egger	13	0.184	0.91	0.80-1.04	

*Abbreviations*: *OC* Ovarian cancer, *OCED* Endometrioid ovarian cancer, *OCCC* Clear cell ovarian cancer, *SOC* Serous ovarian cancer, *MOC* Mucinous ovarian cancer, *IVW* Inverse variance weighted, *WM* Weighted median

#### Ovarian cancer

We found that genetically predicted asparagine was associated with a low risk of OC (OR = 0.65, 95% CI: 0.45-0.95, *P* = 0.024) in the IVW method. The association between asparagine and OC remained stable in the WM method, and the results of the MR Egger method were similar to the primary method. Four metabolites were associated with a high risk of OC: 4-acetamidobutanoate (OR = 1.78, 95% CI: 1.07-2.95, *P* = 0.026), alpha-hydroxyisovalerate (OR = 1.49, 95% CI: 1.08-2.05, *P* = 0.016), 3-(3-hydroxyphenyl)propionate (OR = 1.17, 95% CI: 1.03-1.32, *P* = 0.018),X-13183—stearamide (OR = 1.40, 95% CI: 1.09-1.80, *P* = 0.008). Among them, we found that the *p*-value of 4-acetamidobutanoate for OC was less than 0.05, with the OR values and confidence intervals being close to each other, as verified by the IVW, WM and MR Egger methods, and the causal relationships were all consistent with each other as a salient potential protective factor.

#### Mucinous ovarian cancer

Two metabolites were suggestively associated with MOC using IVW method: 1,5-anhydroglucitol (1,5-AG) (OR = 2.33, 95% CI: 1.17-4.64, *P* = 0.016), ADpSGEGDFXAEGGGVR (OR = 2.52, 95% CI: 1.33-4.77, *P* = 0.005), meanwhile, the results from the WM method were consistent.

#### Clear cell ovarian cancer

In the OCCC analysis with the IVW method, betaine was associated with a reduced risk of it (OR = 0.21, 95% CI: 0.05-0.88, *P* = 0.033), but estrone 3-sulfate was associated with an increased risk (OR = 1.32, 95% CI: 1.00-1.75, *P* = 0.049), their results using the WM method support their respective causal effects.

#### Endometrioid ovarian cancer

In addition, in the analysis of OCED, four metabolites were associated with a high risk of it: 3-(3-hydroxyphenyl)propionate (OR = 1.52, 95% CI: 1.16-2.00, *P* = 0.003), 1,5-anhydroglucitol (1,5-AG) (OR = 2.35, 95% CI: 1.22-4.53, *P* = 0.011), 1-linoleoylglycerophosphoethanolamine (OR = 2.98, 95% CI: 1.24-7.13, *P* = 0.014), ADpSGEGDFXAEGGGVR (OR = 2.35, 95% CI: 1.04-5.29, *P* = 0.040), as for ADpSGEGDFXAEGGGVR, the MR Egger method produced similar estimates (OR = 19.77, 95% CI: 3.22-121.42, *P* = 0.023), though with wider confidence intervals.

#### Serous ovarian cancer

Similarly, in the IVW method, the analysis revealed causal associations between two metabolites and the low risk of SOC: X-13183—stearamide (OR = 1.48, 95% CI: 1.11-1.97, *P* = 0.008), DSGEGDFXAEGGGVR (OR = 0.73, 95% CI: 0.56-0.96, *P* = 0.023), 2-hydroxyhippurate (salicylurate) (OR = 0.93, 95% CI: 0.87-0.99, *P* = 0.028), and their results from the WM method support such a causal effect.

Furthermore, two metabolites were associated with a low risk: arachidonate (20:4n6) (OR = 0.38, 95% CI: 0.16-0.92, *P* = 0.033), stearidonate (18:4n3) (OR = 0.36, 95% CI: 0.15-0.87, *P* = 0.023). We found that 3-(3-hydroxyphenyl) propionate potentially causally related to OCED was the same as OC; 1,5-anhydroglucitol (1,5-AG) and ADpSGEGDFXAEGGGVR potentially causally related to OCED was the same as MOC, and that the analysis estimates were close. The reported OR values in our study are interpreted as changes per 1-SD increase in metabolite levels, aligning with the methodological framework utilized in the research conducted by Wang et al. [32]. However, FDR correction for these *P* values did not show significant confirmative association, and the results as suggestive causal associations (Table 2, specific information can be found at Supplement Table S5).

No evidence of pleiotropy or heterogeneity was found in the robust causal relationships listed above (Table 3), which suggested that the main result of the IVW method in our study could provide reliability for causal effect with low heterogeneity. In addition, the statistical power of causal inference calculated by the IVW method reached 1 for all metabolites except one, which was 0.85, with a Type I error rate of 0.05 (Supplement Table S6). Leave-one-out analysis indicated that none of the associations were driven solely by a single SNP, suggesting a stable result (Supplementary Figures 1 and 2).

**Table 3:** The results of heterogeneity testing and pleiotropy testing for candidate blood metabolites and several OC phenotypes

Outcome	Exposure	Nsnp	Heterogeneity Q	Pval	Pleiotropy intercept	Pval
MOC	ADpSGEGDFXAEGGGVR	7	5.416	0.49	-0.021	0.66
MOC	1,5-anhydroglucitol (1,5-AG)	31	29.820	0.47	-0.018	0.26
OC	X-13183--stearamide	11	7.638	0.66	-0.004	0.79
OC	alpha-hydroxyisovalerate	17	10.200	0.86	-0.005	0.71
OC	3-(3-hydroxyphenyl)propionate	11	9.816	0.46	0.001	0.96
OC	asparagine	46	45.809	0.44	0.006	0.19
OC	4-acetamidobutanoate	43	56.405	0.07	-0.015	0.06
OCCC	betaine	24	28.526	0.20	0.024	0.39
OCCC	estrone 3-sulfate	13	14.069	0.30	0.015	0.61
OCED	3-(3-hydroxyphenyl)propionate	11	4.949	0.89	-0.036	0.23
OCED	1,5-anhydroglucitol (1,5-AG)	31	17.186	0.97	0.003	0.83
OCED	1-linoleoylglycerophosphoethanolamine	11	10.410	0.41	-0.017	0.58
OCED	stearidonate (18:4n3)	11	13.599	0.19	0.038	0.30
OCED	arachidonate (20:4n6)	20	19.316	0.44	0.018	0.20
OCED	ADpSGEGDFXAEGGGVR	7	10.734	0.10	-0.102	0.06
SOC	X-13183--stearamide	11	9.275	0.51	-0.005	0.76
SOC	DSGEGDFXAEGGGVR	13	8.823	0.72	0.020	0.27
SOC	2-hydroxyhippurate (salicylurate)	13	7.945	0.79	0.004	0.77

*Abbreviations*: *OC* Ovarian cancer, *OCED* Endometrioid ovarian cancer, *OCCC* Clear cell ovarian cancer, *SOC* Serous ovarian cancer, *MOC* Mucinous ovarian cancer

### Reverse Mendelian randomization

To validate whether the observed blood metabolite levels were influenced by OC risk, we conducted a reverse MR analysis, treating OC as the exposure and blood metabolites as the outcome. The results did not show evidence of OC impacting blood metabolite levels (Table 4).

**Table 4:** The reverse Mendelian randomization analysis results: causal relationships between several OC phenotypes and candidate blood metabolites

Outcome	Exposure	method	nsnp	OR(95%CI)	pval
**Amino acid**					
4-acetamidobutanoate	OC	IVW	6	1.00 (0.98-1.02)	0.932
		WM	6	1.00 (0.98-1.02)	0.878
		MR Egger	6	1.01 (0.96-1.06)	0.710
alpha-hydroxyisovalerate	OC	IVW	6	1.00 (0.97-1.04)	0.931
		WM	6	1.01 (0.97-1.05)	0.565
		MR Egger	6	1.05 (0.96-1.14)	0.366
asparagine	OC	IVW	6	1.01 (0.99-1.02)	0.550
		WM	6	1.00 (0.98-1.03)	0.662
		MR Egger	6	1.00 (0.96-1.05)	0.919
betaine	OCCC	Wald ratio	1	1.00 (0.97-1.03)	0.937
3-(3-hydroxyphenyl)propionate	SOC	IVW	10	0.97 (0.90-1.04)	0.420
		WM	10	0.99 (0.90-1.10)	0.861
		MR Egger	10	0.95 (0.69-1.32)	0.784
**Carbohydrate**					
1,5-anhydroglucitol (1,5-AG)	MOC	IVW	3	0.99 (0.98-1.00)	0.153
		WM	3	0.99 (0.97-1.01)	0.210
		MR Egger	3	1.04 (0.74-1.46)	0.851
1,5-anhydroglucitol (1,5-AG)	OCED	IVW	2	0.99 (0.96-1.02)	0.496
**Lipid**					
estrone 3-sulfate	OCCC	Wald ratio	1	0.97 (0.82-1.14)	0.722
arachidonate (20:4n6)	OCED	IVW	2	0.99 (0.93-1.05)	0.739
1-linoleoylglycerophosphoethanolamine	OCED	IVW	2	1.00 (0.96-1.04)	0.964
stearidonate (18:4n3)	OCED	IVW	2	1.01 (0.97-1.05)	0.670
X-13183--stearamide	SOC	IVW	7	0.99 (0.94-1.03)	0.561
		WM	7	0.98 (0.92-1.04)	0.458
		MR Egger	7	0.89 (0.72-1.09)	0.311
**Peptide**					
ADpSGEGDFXAEGGGVR	OCED	IVW	2	0.95 (0.89-1.01)	0.121
DSGEGDFXAEGGGVR	SOC	IVW	7	1.01 (0.96-1.06)	0.807
		WM	7	1.00 (0.94-1.07)	0.911
		MR Egger	7	0.88 (0.70-1.10)	0.323
ADpSGEGDFXAEGGGVR	MOC	IVW	3	1.02 (0.97-1.07)	0.469
		WM	3	0.99 (0.95-1.04)	0.758
		MR Egger	3	0.52 (0.23-1.19)	0.367
**Xenobiotics**					
2-hydroxyhippurate (salicylurate)	SOC	IVW	7	0.99 (0.85-1.17)	0.938
		WM	7	0.91 (0.74-1.13)	0.412
		MR Egger	7	1.07 (0.51-2.23)	0.865

*Abbreviations*: *OC* Ovarian cancer, *OCED* Endometrioid ovarian cancer, *OCCC* Clear cell ovarian cancer, *SOC* Serous ovarian cancer, *MOC* Mucinous ovarian cancer, *IVW* Inverse variance weighted, *WM* weighted median

### Metabolic pathway analysis

Pathway analysis identified 5 significant metabolic pathways. The results indicated that the pathways "caffeine metabolism," "arginine biosynthesis," and "citrate cycle (TCA cycle)" were associated with the development of MOC. Additionally, the pathways "caffeine metabolism" and "alpha-linolenic acid metabolism" were associated with the onset of OCED (Table 5).

**Table 5:** Significant metabolic pathways involved in different OC phenotypes

Traits	Metabolic pathways	Total	Hits	Expected	*P*-value
MOC	Caffeine metabolism	10	2	0.019	0.019
MOC	Arginine biosynthesis	14	1	0.027	0.027
MOC	Citrate cycle (TCA cycle)	20	1	0.039	0.038
OCED	Caffeine metabolism	10	1	0.039	0.038
OCED	alpha-Linolenic acid metabolism	13	1	0.050	0.049

*Abbreviations*: *OC* Ovarian cancer, *OCED* Endometrioid ovarian cancer, *OCCC* Clear cell ovarian cancer *SOC* Serous ovarian cancer, *MOC* Mucinous ovarian cancer

## Discussion

In this study, we identified 8 genetically determined metabolites as potential risk factors, and 6 as potential cancer risk reducers. Additionally, pathway enrichment analysis pinpointed four crucial metabolic pathways. To our knowledge, this is the first MR study that assesses the causal relationship between genetically determined metabolites and different subtypes of OC. Furthermore, we have conducted reverse validation of our results, which revealed no causal relationship, eliminating biases related to reverse causation and reinforcing the robustness of our primary MR findings.

In the present study, we identified suggestive causal associations for 4-acetamidobutanoate, alpha-hydroxyisovalerate, 3-(3-hydroxyphenyl)propionate, X-13183-stearamide, 1,5-anhydroglucitol (1,5-AG), ADpSGEGDFXAEGGGVR, estrone 3-sulfate, and 1-linoleoylglycerophosphoethanolamine associated with a high risk of developing OC. To our knowledge, previous research related to these 8 metabolites in association with OC has been limited. Among the 3 amino acids, 4-acetamidobutanoate is a derivative of γ-aminobutyric acid (GABA) [33]. In recent years, GABA has been shown to be associated with promoting the proliferation of pancreatic cancer [34]. Adding GABA to cell culture media promoted the proliferation of pancreatic cancer cells expressing GABRP [35], which is somewhat consistent with our study. Notably, in a study on unique metabolomic characteristics related to cirrhosis mortality [36], 4-acetamidobutanoate significantly predicted mortality. It's reported that in patients with acute kidney injury (AKI), 4-acetamidobutanoate increased 12-fold [37], and its levels significantly increased in patients with morbid hypertension [38]. Similarly, alpha-hydroxyisovalerate, an organic acid related to branched-chain amino acid metabolism, has been linked with liver injury [39], diabetic nephropathy [40], and Maple Syrup Urine Disease [41]. These findings might help in predicting the prognostic features of OC patients.

X-13183-stearamide, estrone 3-sulfate, and 1-linoleoylglycerophosphoethanolamine are all lipid metabolic factors. Among them, estrone 3-sulfate (E1S) is a naturally occurring endogenous steroidal compound, classified under estrogen esters and estrogen conjugates [42]. E1S has associations with multiple transport proteins and plays a pivotal role in the uptake and release of drugs and endogenous substances [43]. It can be taken up by tumor cells through transport protein mediation, and upon cleavage by steroid sulfatase, eventually activating ERs and promoting tumor growth [44]. This aligns with our research findings. 1-linoleoylglycerophosphoethanolamine is a vital member of the phosphatidylethanolamine (PE) family [45], and might serve as an intermediary in the primary synthesis route of PE — the CDP-Ethanolamine Pathway [46]. Studies have shown that this substance plays a part in the development of preeclampsia during pregnancy [45] and colorectal cancer [47]. PE family are critical determinants of protein structure and function [46]. Aberrant levels of 1-linoleoylglycerophosphoethanolamine might lead to disruptions in the PE synthesis pathway, subsequently resulting in pathological conditions.

We identified suggestive causal associations for 6 metabolic products that inhibit OC development. Among them, asparagine is an essential natural amino acid that healthy cells utilize to maintain function and proliferation [48]. Its role as a targeted anticancer amino acid aligns with our findings [48]. Betaine, another vital amino acid, has been shown to have chronic disease prevention potential [49]. Research indicates that the content of betaine is higher in gluten-free cereals and products, suggesting that this result might provide evidence for dietary guidance for patients.

Pathway enrichment analysis revealed four significant metabolic pathways, with three linked to MOC onset and two to OCED onset. The potential impact of caffeine metabolism on the risk of MOC and OCED may be attributed to how caffeine and its metabolic pathways affect the levels of sex hormones[50, 51]. Coffee intake, as shown in a large retrospective study, reduces susceptibility to colon cancer[52], possibly due to metabolites formed via liver cytochrome P450 enzyme system metabolism [53]. These studies align with our findings, suggesting that intervening in caffeine metabolism could potentially reduce the risk of cancer onset.

Arginine synthesis and metabolic pathways maintain nitrogen balance and protein synthesis processes, providing cells with necessary substances and energy, supporting rapid proliferation and survival of cancer cells [54]. Arginine can be degraded by enzymes in macrophages to produce urea and L-ornithine, which might inhibit the function of T cells [55]. This mechanism might help cancer cells evade immune clearance, increasing the risk of tumor onset. It's worth mentioning that our results are consistent with the above, suggesting it is a potential MOC risk factor.

The relationship between the citrate cycle (TCA cycle) and MOC was also observed. The citrate cycle, a primary cellular energy production pathway, is implicated in cancer biology by regulating glycolysis [56], immune responses [57], and affecting tumor cell activity [58]. Citrate synthase (CS) is one of the key enzymes in the TCA cycle. Silencing CS leads to proliferation defects in SKOV3 cells, inhibits invasion and migration, and increases chemosensitivity, indicating the citrate cycle pathway might affect the progression and drug resistance in OC [59].

Moreover, Our research results also suggest that the metabolism of α-linolenic acid may be one of the protective pathways against the onset of OC. Numerous studies have confirmed α-linolenic acid, an essential polyunsaturated fatty acid, may regulate tumor proliferation, migration, and invasion by controlling inflammation-related cytokine secretion and cellular signal pathways [60]. Eicosapentaenoic Acid (EPA) and Docosahexaenoic acid (DHA) are both metabolites of α-linolenic acid and have shown significant anti-ovarian cancer effects [61]. However, the impact of this pathway on OC and its mechanisms warrant further study.

Regrettably, we must acknowledge that our findings do not pass the multiple testing correction. The reasons for these outcomes might include the following factors. Firstly, OC is a complex disease likely influenced by multiple factors. Metabolic disorders are just one aspect and are not specific to the pathogenesis of OC. They might manifest as abnormalities in the internal environment during the onset of OC. MR studies are primarily utilized to deduce causal relationships between exposures and outcomes. Therefore, abnormalities in serum metabolic factors, may indicative of aberrant metabolic environment during OC rather than merely representing a simple causal relationship.

Secondly, while individual intermediate metabolic products may exert only minor or indirect effects on the onset of OC, their combined impact could be significantly more substantial, resembling the effect of polygenic risk scores in complex traits.

The third potential factor may be attributed to individual variations in metabolic factors. While genetic elements significantly shape distinct metabolite profiles across various populations, it is imperative to recognize the substantial variability of serum metabolic factors among individuals. Influences such as sex, lifestyle, and dietary habits contribute to these disparities. For instance, sphingolipid depletion, known to impede vitamin absorption, is closely associated with vegetable intake [62]. A Study highlights disparities in blood sphingolipid levels between traditional and non-traditional lifestyles in Swedish populations [63]. Moreover, the metabolic environment in OC fluctuates across different disease stages [64], and singular sampling and measurement may not accurately capture the patient's dynamic metabolic changes. Due to the limitations imposed by the original data, we were unable to categorize patients more precisely, pointing to the need for more nuanced research in this area.

Lastly, the research methodology may have also influenced these findings. Although MR is designed to mitigate the effects of confounding variables, potential uncontrolled confounders, including undetected genetic variations, might still exist.

Although we did not demonstrate a definitive causal effect of GDMs on OC and other subtypes, we believe these indicative results do not repudiate the role of blood metabolites in the pathogenesis of OC. An increasing number of observational studies indicate metabolic abnormalities in cancer patients compared to healthy controls, suggesting potential guidance for targeted treatment in OC patients [65–67] For instance, beyond the indicative results we have already explained, our research discovered the potential protective role of 1,5-Anhydroglucitol (1,5-AG) as a potential protective factor against MOC and OCED. The reduced levels of 1,5-AG typically reflect increased blood glucose levels [68], a known risk factor for OC [69]. Furthermore, we observed that treatment alters measurable circulating metabolites and lipoprotein subfractions, potentially serving as biomarkers for recurrence risk [70]. A metabolomic analysis involving 35 patients with EOC demonstrated that changes in serum metabolic factors could help predict EOC recurrence [71]. Thus, while a precise causal relationship of individual metabolic products was not detected, they may still represent risk factors and key intermediaries in the development of OC.

Additionally, we observed that the study by Feng et al. also examined the relationship between GDMs and OC [72]. Interestingly, our study found different associations, likely due to the different thresholds used for selecting IVs. These varying thresholds led to the inclusion of different genetic variants in our analysis. This discrepancy highlights the need to further explore the impact of diverse IV selection criteria, as they may uncover distinct biological relationships. Future research could beneficially focus on how these criteria affect MR analyses, thereby enriching our understanding of the genetic influences on metabolites and their role in the etiology of OC.

## Limitations and future directions

Those preliminary findings offer a direction for further exploration. We advocate for enhanced screening of populations exhibiting metabolic abnormalities, recognizing its vital role in the clinical prevention and prognosis of OC. We also recommend longitudinal follow-up of patients to delve deeper into biomarkers of cancer recurrence.

This study presents several limitations. Firstly, based on the analysis we have previously conducted, the absence of sex-specific instruments and genetic heterogeneity may contribute to confounders. The accuracy of MR analyses heavily relies on the interpretation of exposure IVs, expanding the sample size and enhancing metabolome measurements are imperative. Lastly, our study's focus on the European population limits its generalizability, necessitating further validation across diverse populations. Future research might need to categorize and describe phenotypes more precisely, and refine statistical models to reduce bias, thereby enhancing the accuracy and generalizability of the findings.

## Conclusion

This MR study identified 18 suggestive causal relationships involving 14 known metabolites and determined four crucial metabolic pathways potentially related to the pathophysiology of OC. These findings enhance our understanding of OC's pathogenesis, including its various subtypes, and could inform the development of more effective management strategies in clinical settings. However, the lack of strong corroborative evidence necessitates further research to both confirm these relationships and extend these results to understand their clear implications in the context of OC treatment and prognosis.

### Supplementary Information

**Additional file 1.** 

**Additional file 2:** **Supplementary Figure S1. **Forest plots for the Mendelian randomization (MR) leave one out analysis of the significant and nominal significant results.

**Additional file 3:** **Supplementary Figure S2. **Scatter plots of the metabolites-SNP associations (x-axis) versus the OC-SNP associations (y-axis) were shown.

**Additional file 4: Table S1.** Classification and detailed information of 486 blood metabolites.

**Additional file 5: Table S2.** Detailed information on instrumental variables for candidate blood metabolites (*P*<5e-8).

**Additional file 6: Table S3.** Detailed information on instrumental variables for candidate blood metabolites.

**Additional file 7: Table S4.** The total R2 and the median F-statistic (and range) for each metabolite.

**Additional file 8: Table S5.** 

**Additional file 9: Table S6.** Power calculations for Mendelian Randomization.

## Data Availability

The datasets for the genome-wide association study (GWAS) summary statistics can be found in the GWAS Catalog (https://gwas.mrcieu.ac.uk/, accessed on 01 September 2023).
