# Supplementary material for: Exploring the causal role of multiple metabolites on ovarian cancer: a two sample Mendelian randomization study
Source: J Ovarian Res. 2024 Jan 23;17:22. doi: 10.1186/s13048-023-01340-w (PMC10804794; doi:10.1186/s13048-023-01340-w)

**Supplementary Figure S1.** Forest plots for the Mendelian randomization (MR) leave one out analysis of the significant and nominal significant results.

Annotation: Within each panel, the black points represent the causal estimate of association between a specific exposure and target gynecological cancers after discarding each SNP in turn. Red points represent the overall causal estimate using the random-effects inverse variance weighted. Horizontal lines denote 95% confidence intervals.


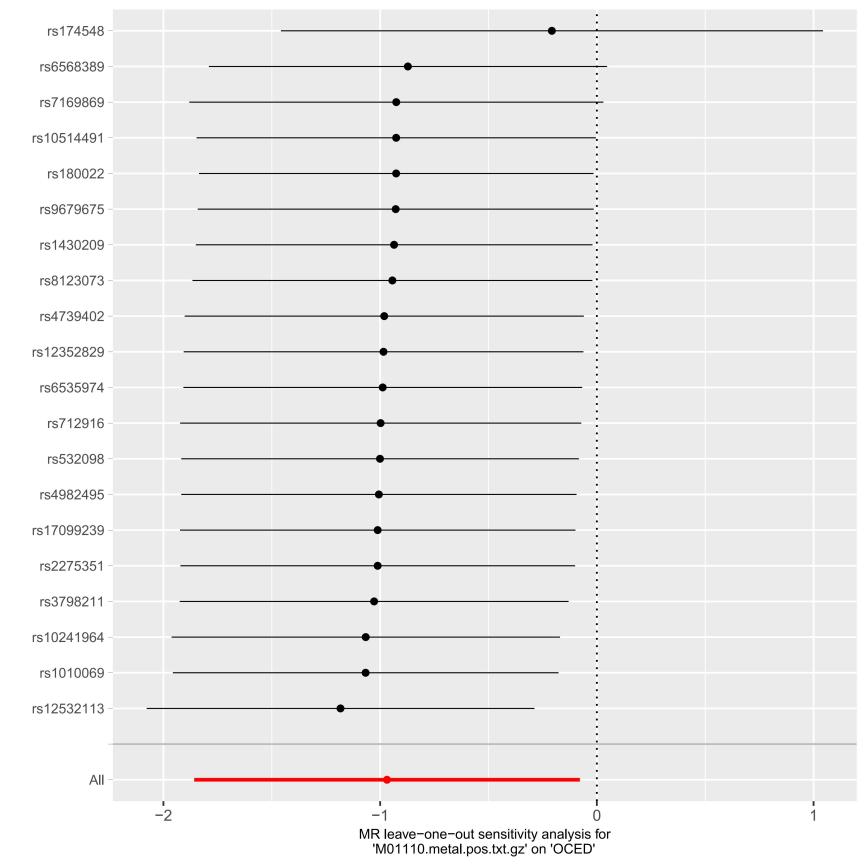


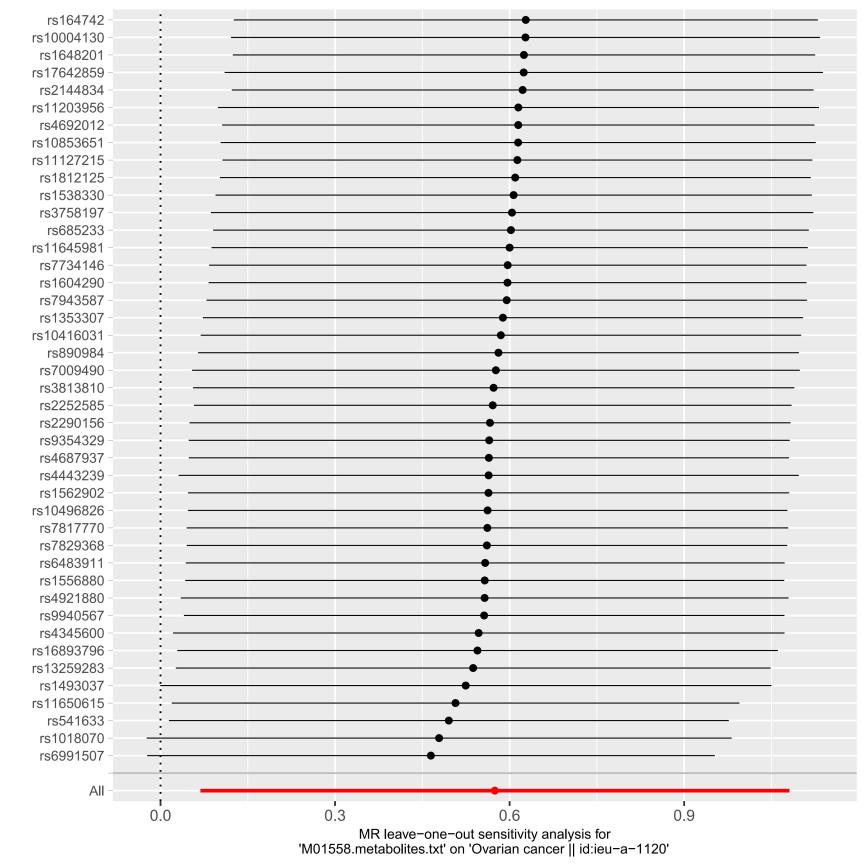

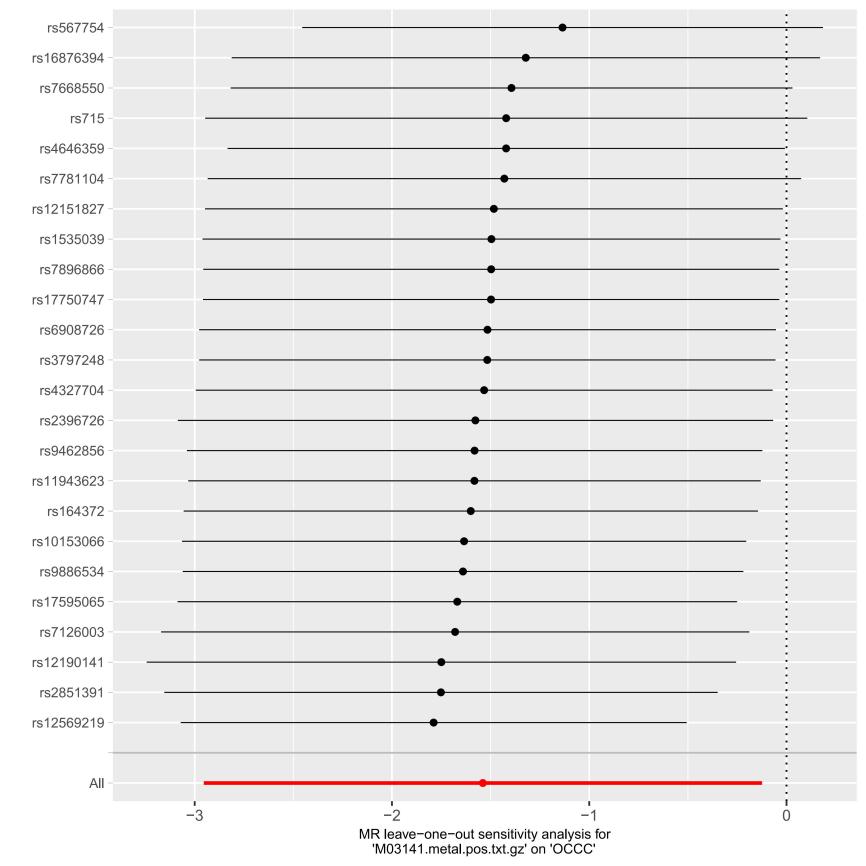


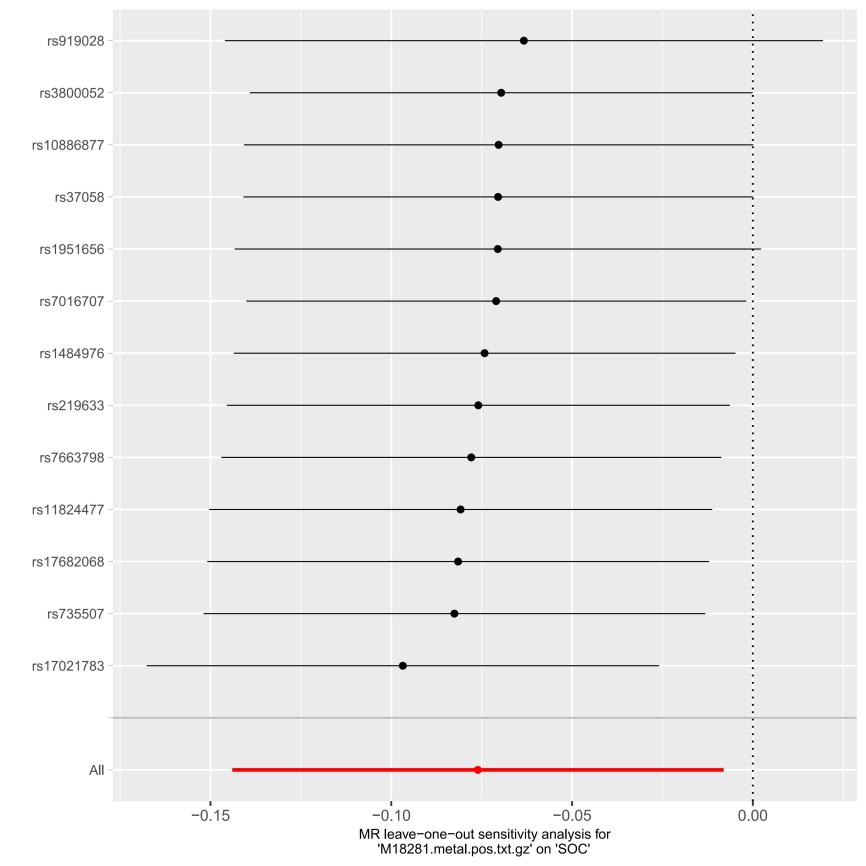

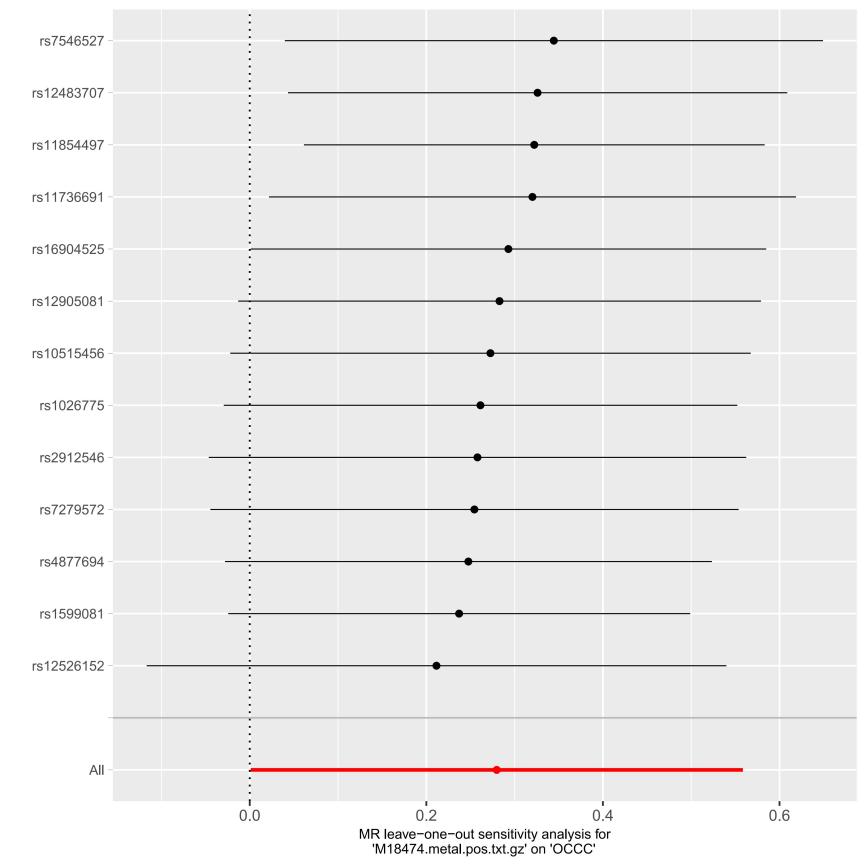


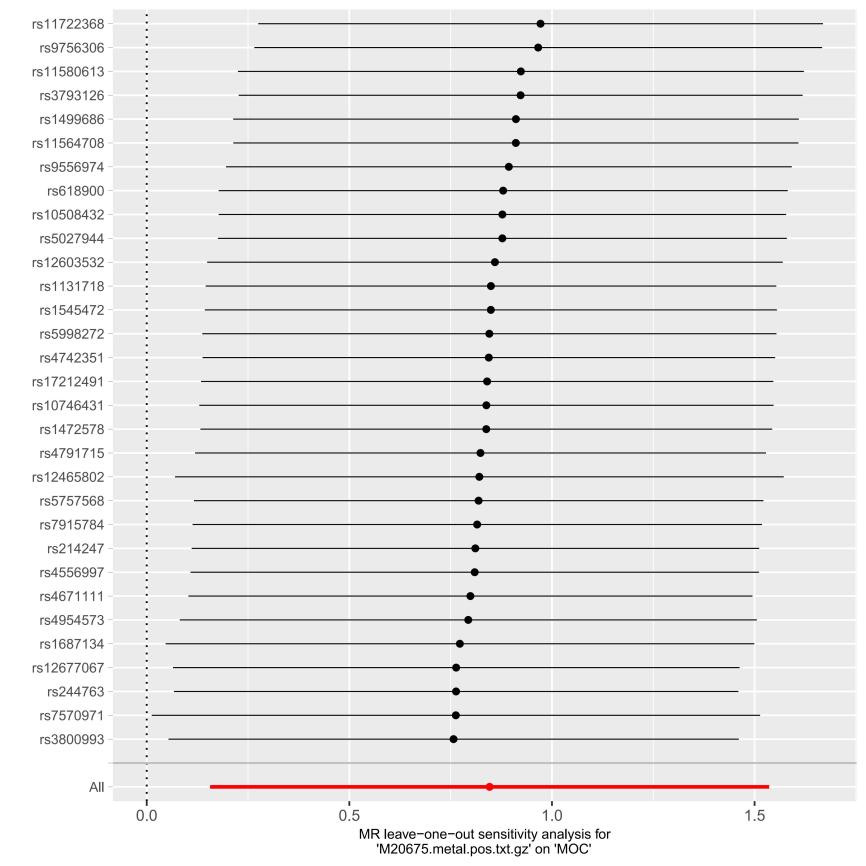

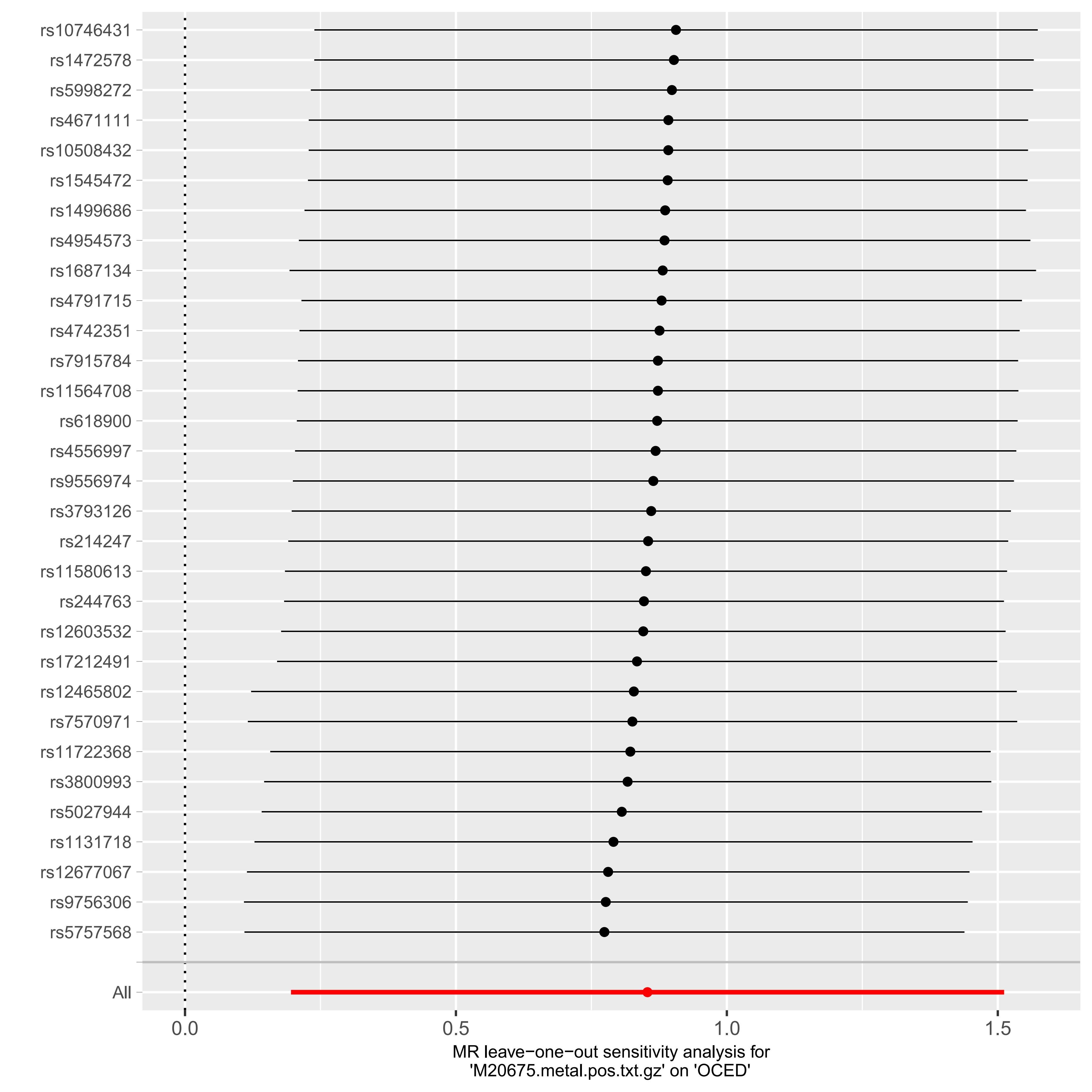


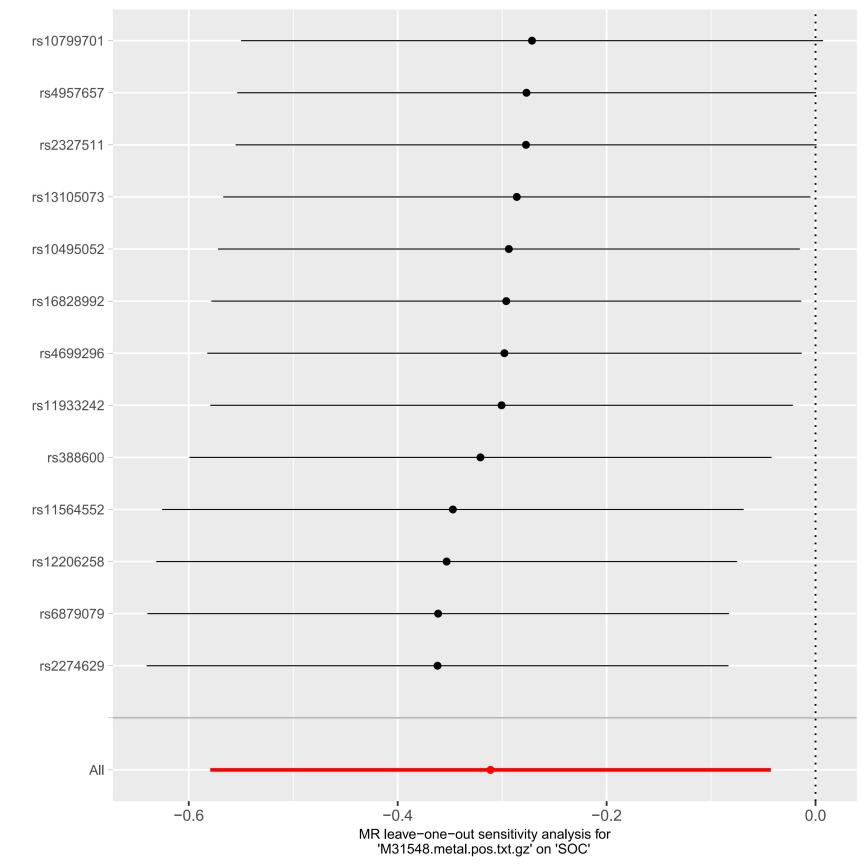

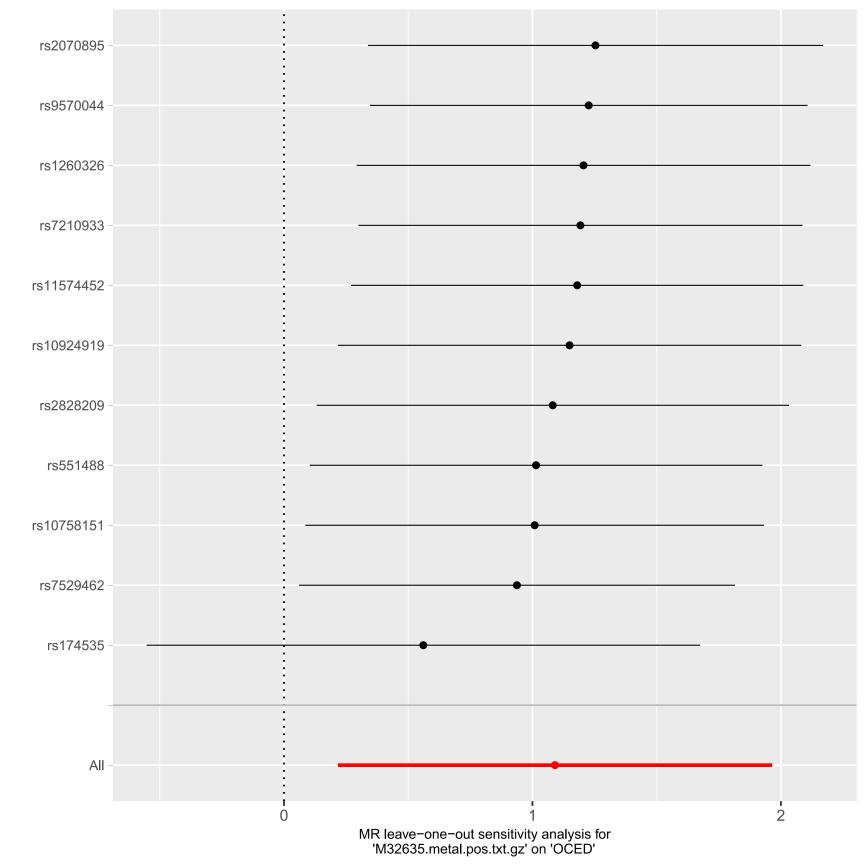


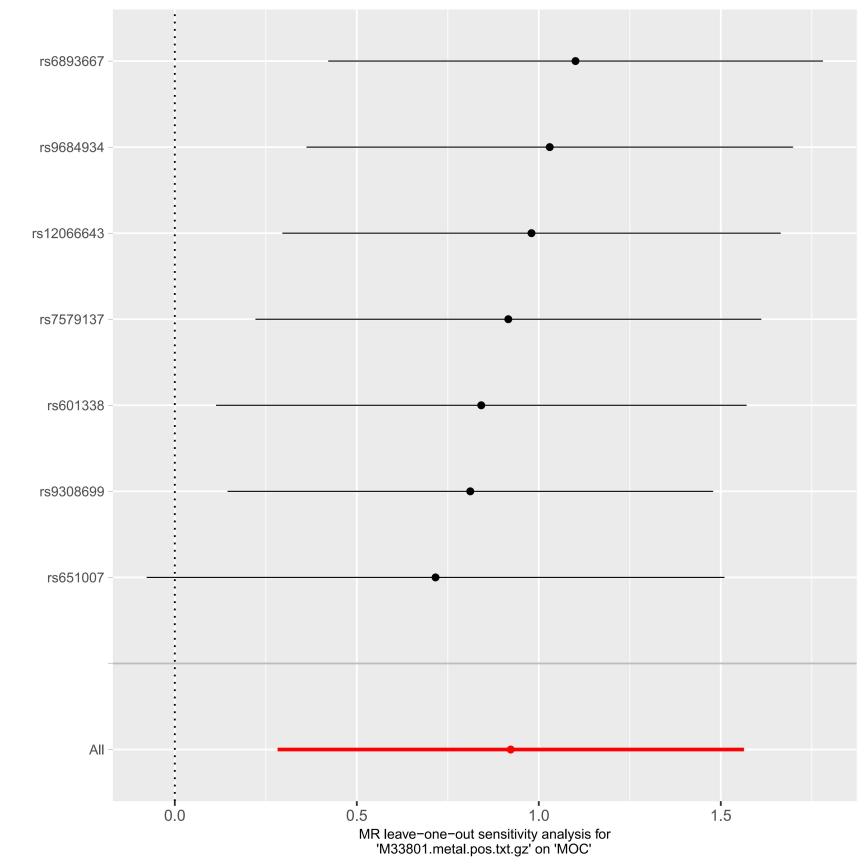

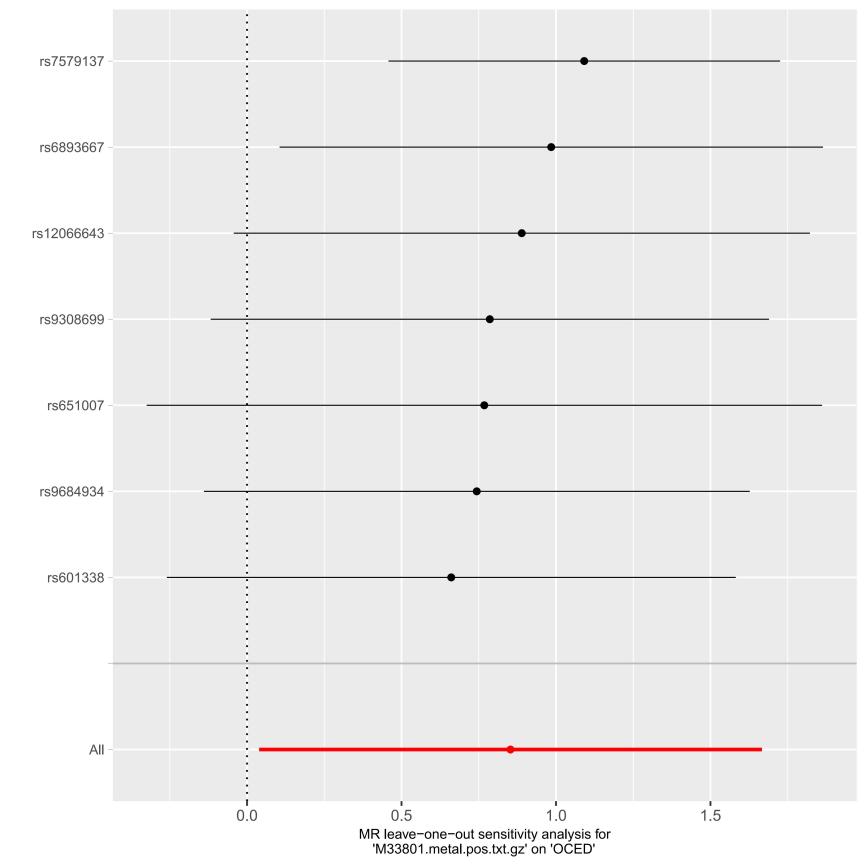


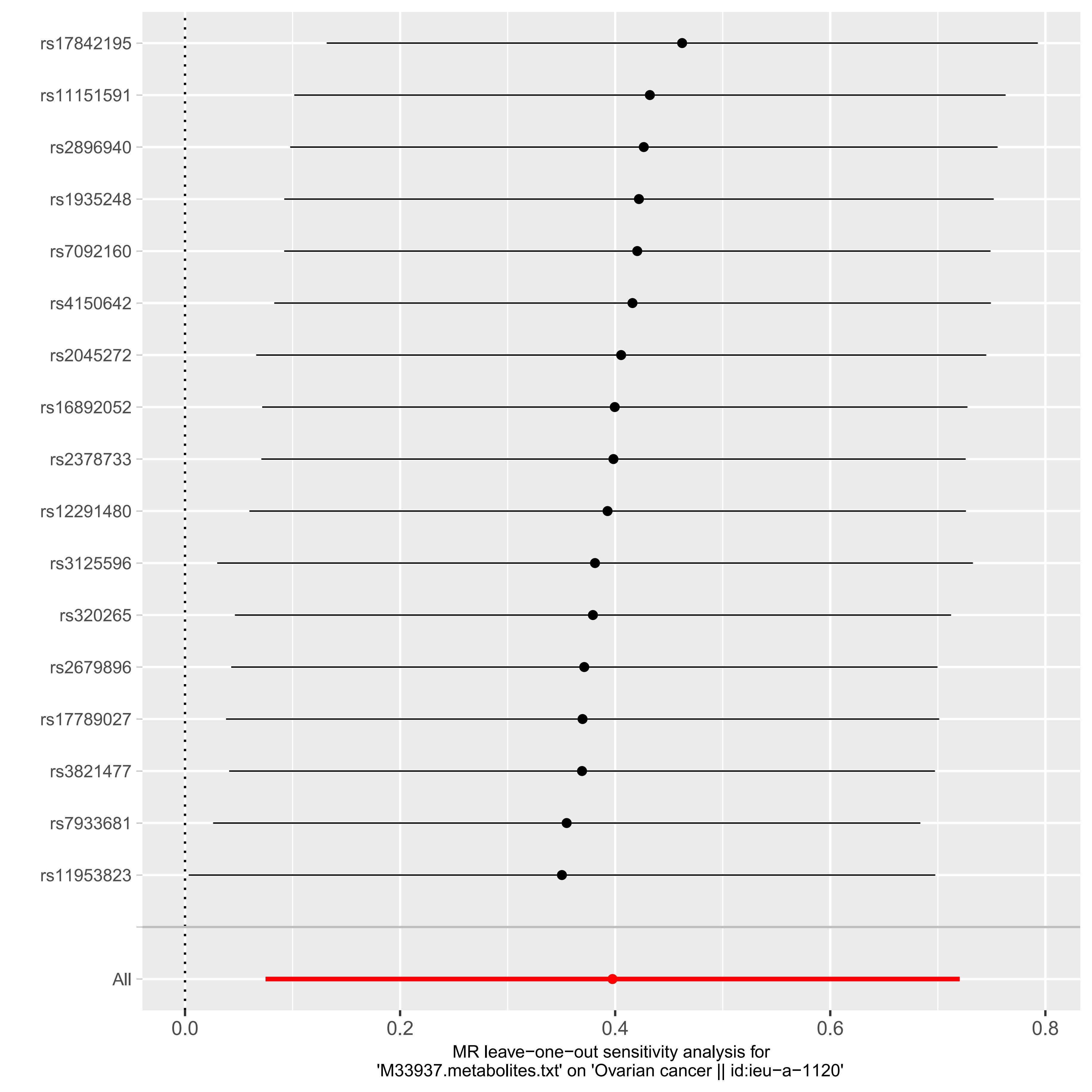

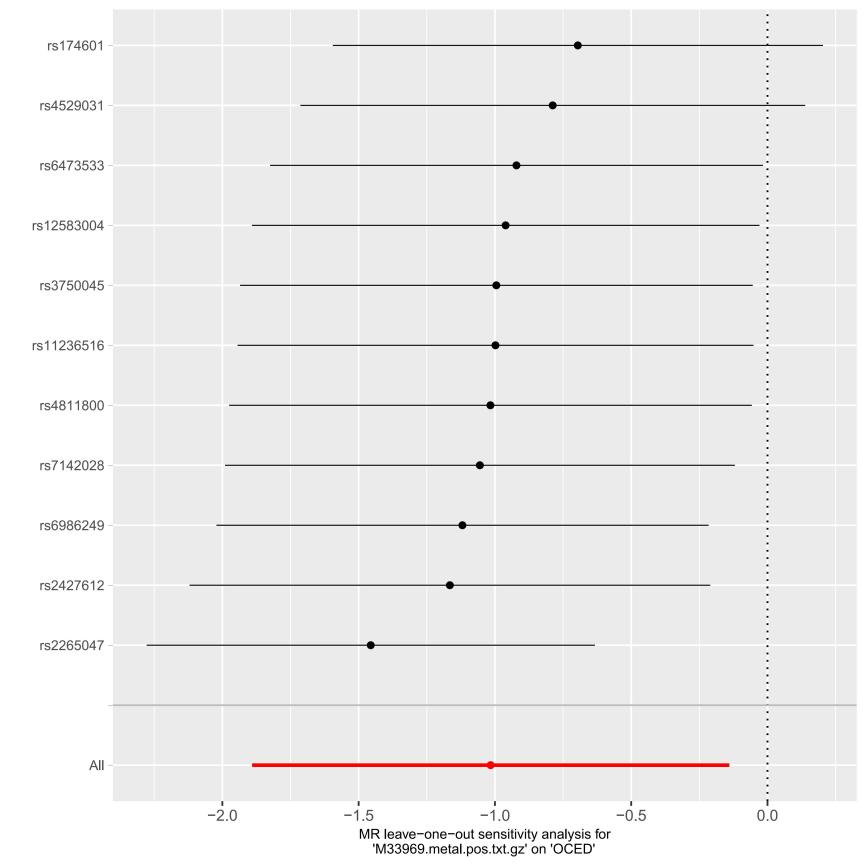


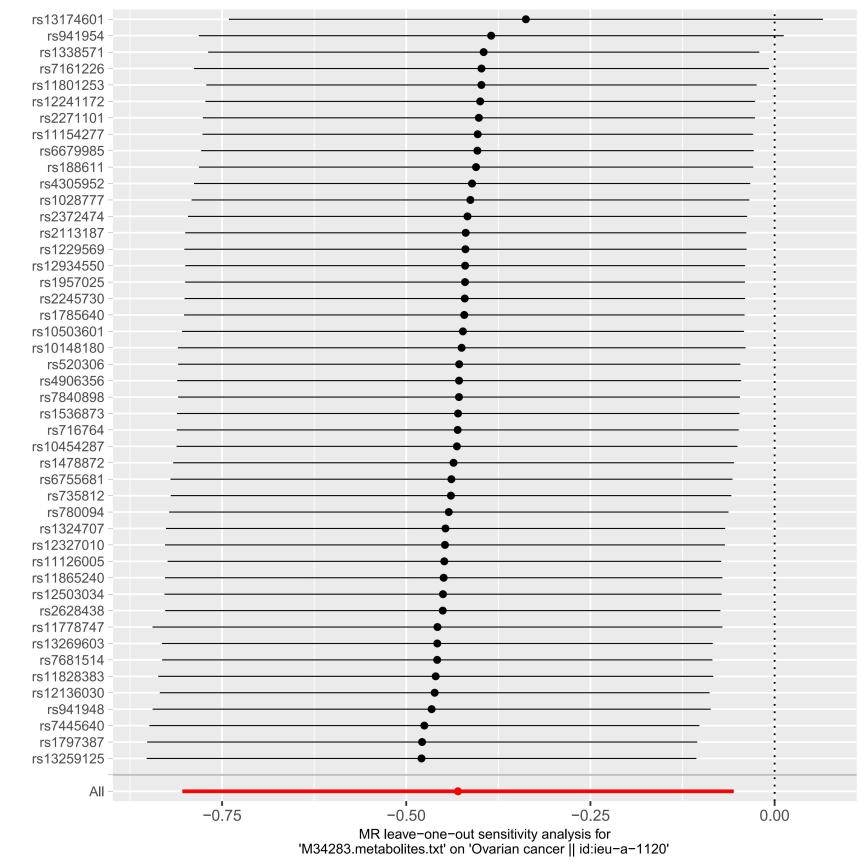

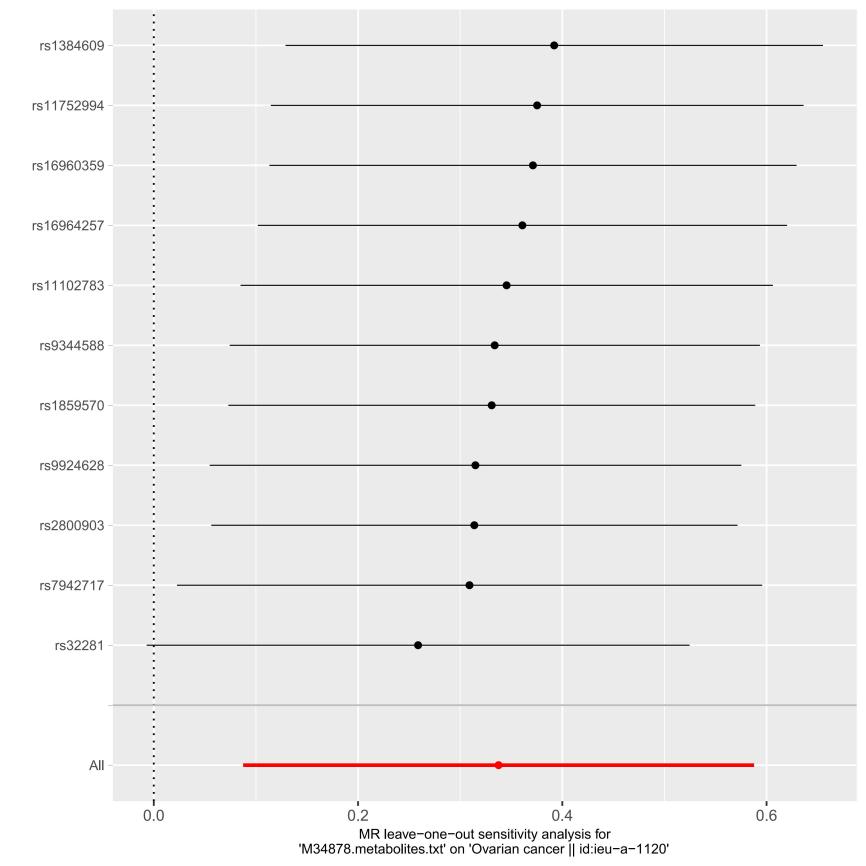


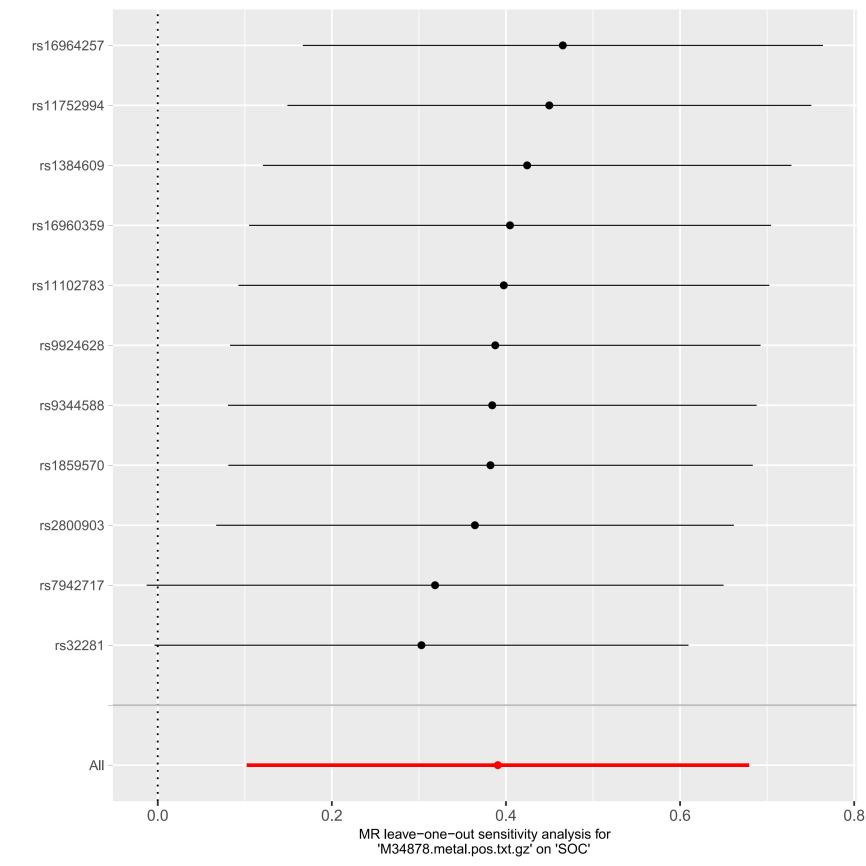

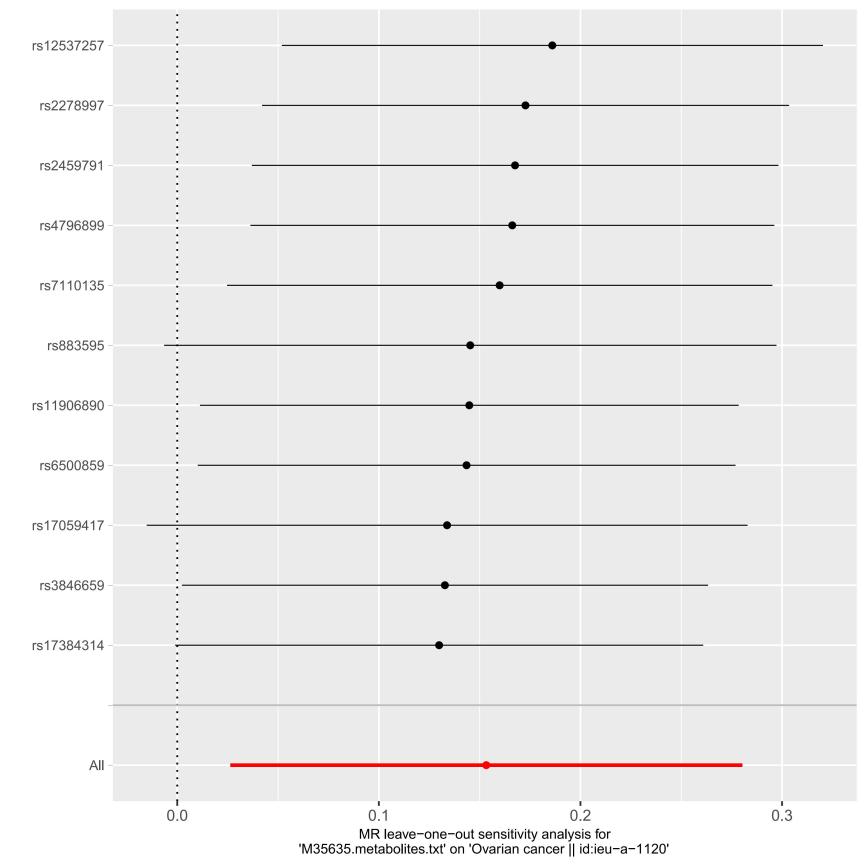


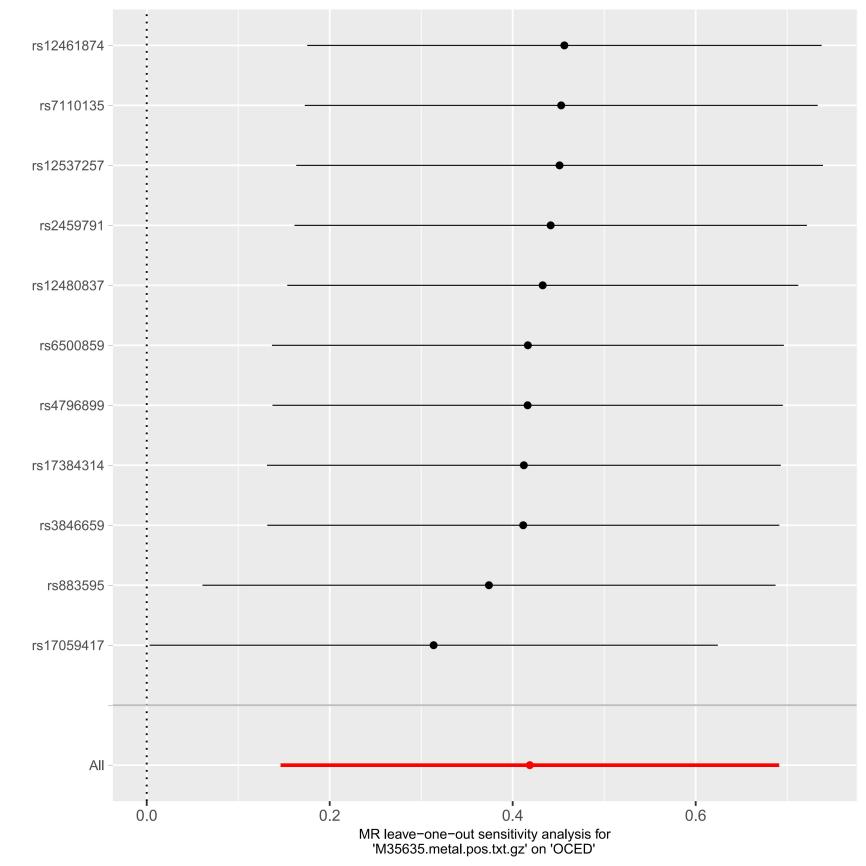

Supplement: Supplementary file 2 — Additional file 2: Supplementary Figure S1. Forest plots for the Mendelian randomization (MR) leave one out analysis of the significant and nominal significant results. [file 13048_2023_1340_MOESM2_ESM.docx]
