# Supplementary material for: Exploring the causal role of multiple metabolites on ovarian cancer: a two sample Mendelian randomization study
Source: J Ovarian Res. 2024 Jan 23;17:22. doi: 10.1186/s13048-023-01340-w (PMC10804794; doi:10.1186/s13048-023-01340-w)

**Supplementary Figure S2.** Scatter plots of the metabolites-SNP associations (x-axis) versus the OC-SNP associations (y-axis) were shown

Annotation: horizontal and vertical lines showing 95 % confidence intervals for each association. The lines that move obliquely upward from left to right show a positive causal direction between the taxa.


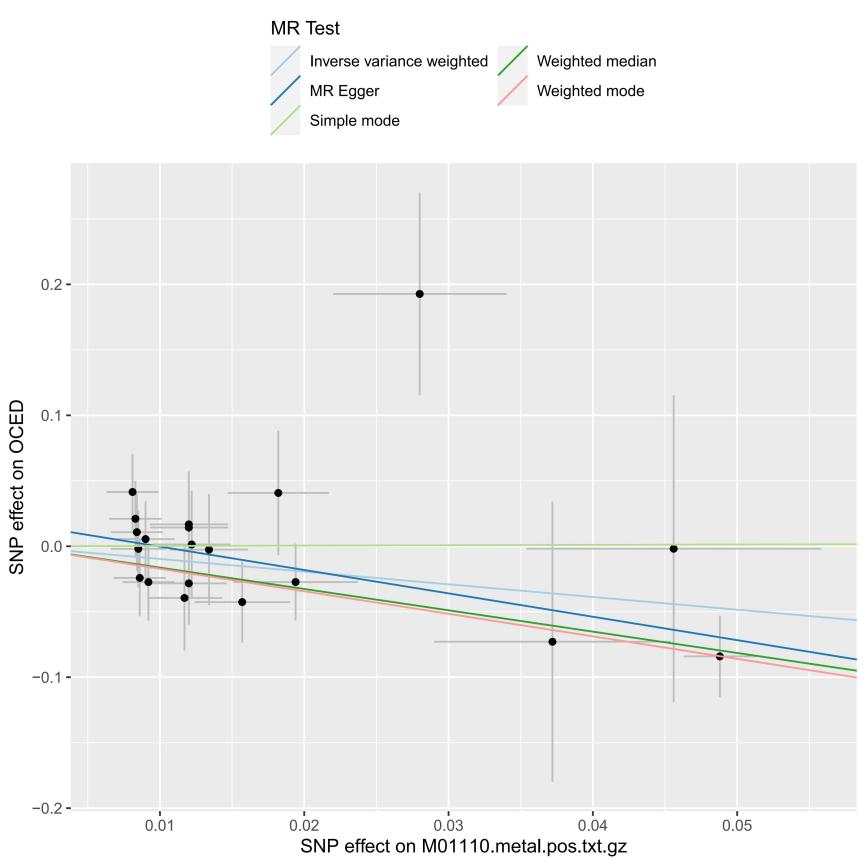


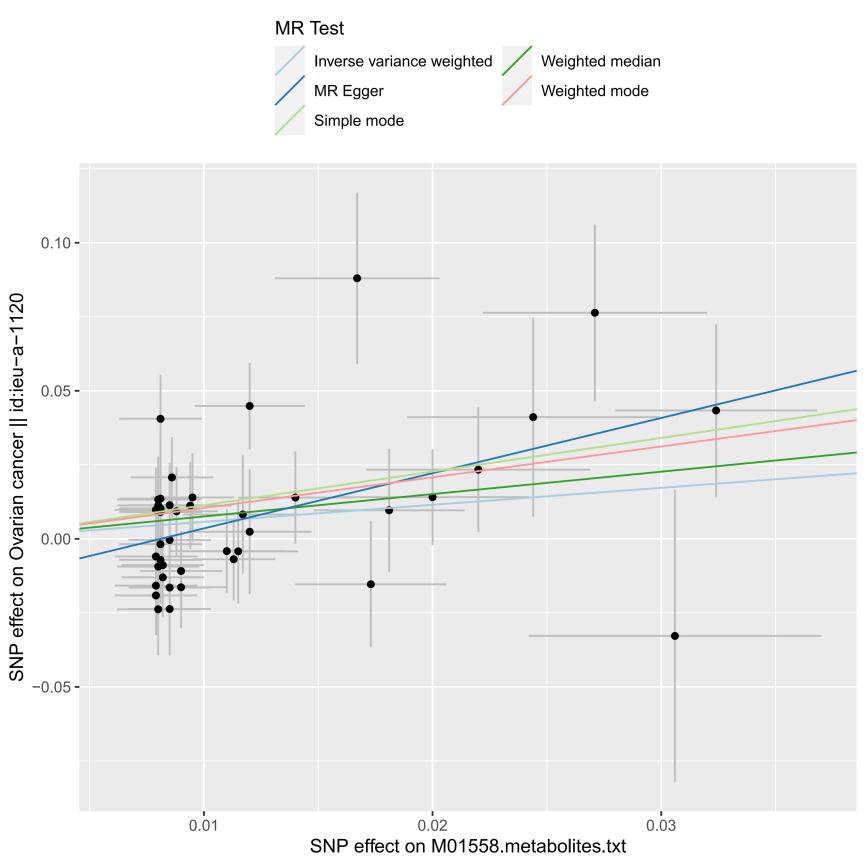

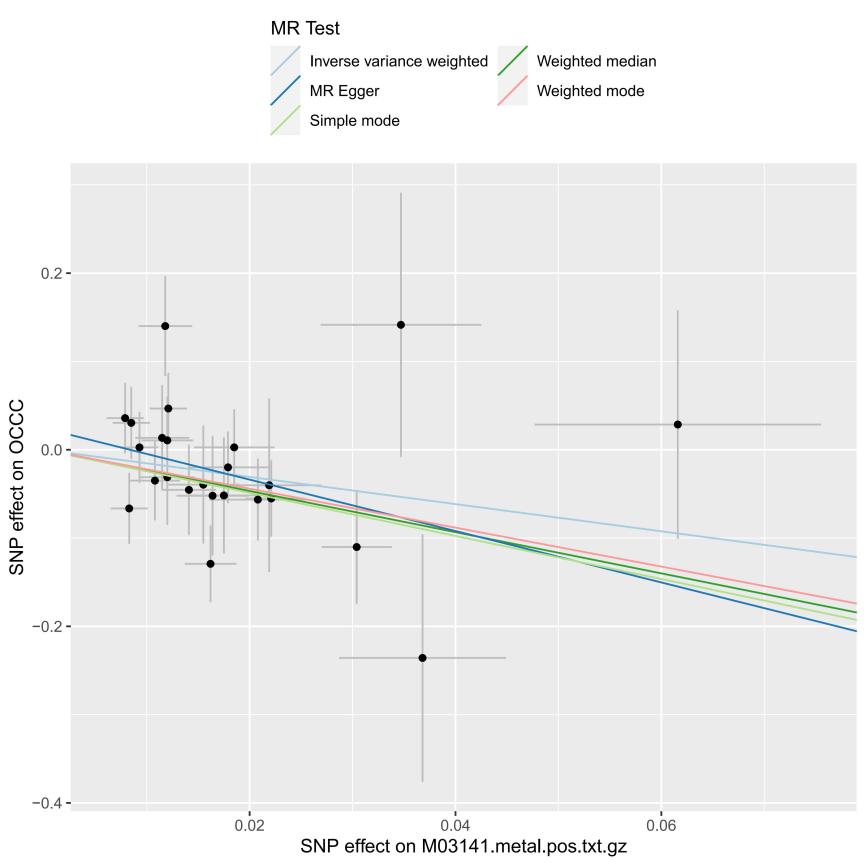


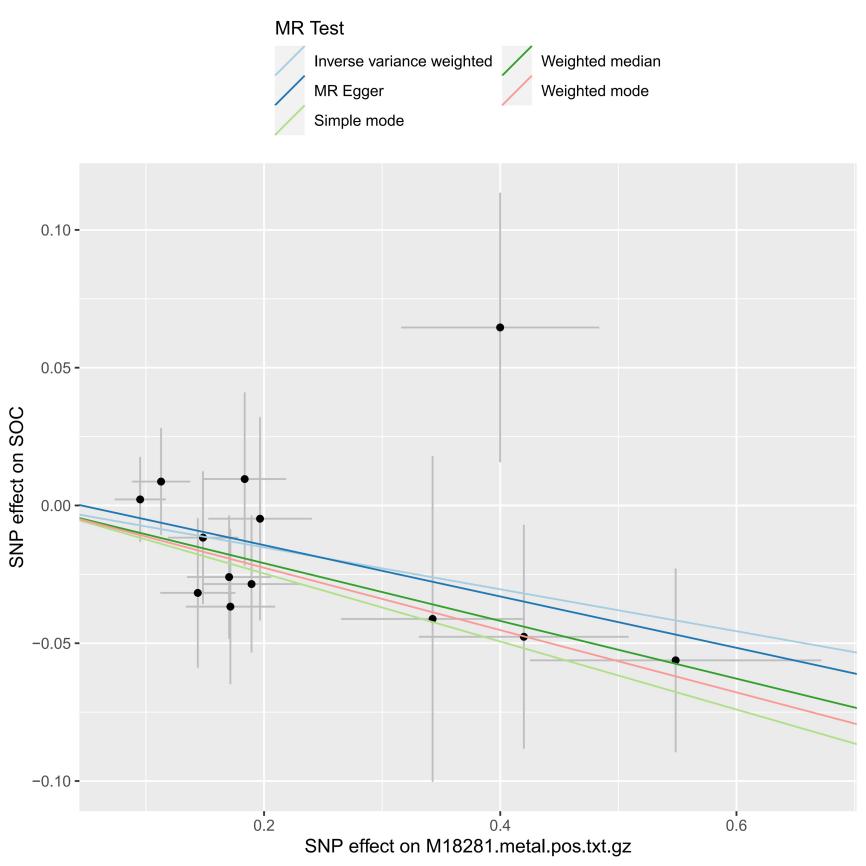

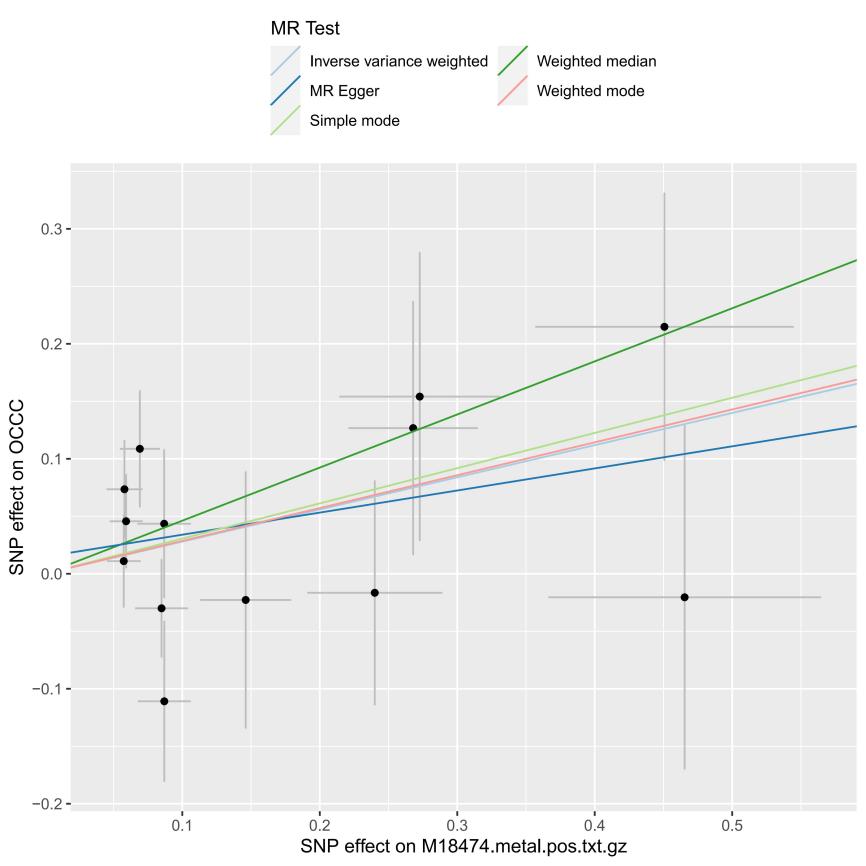


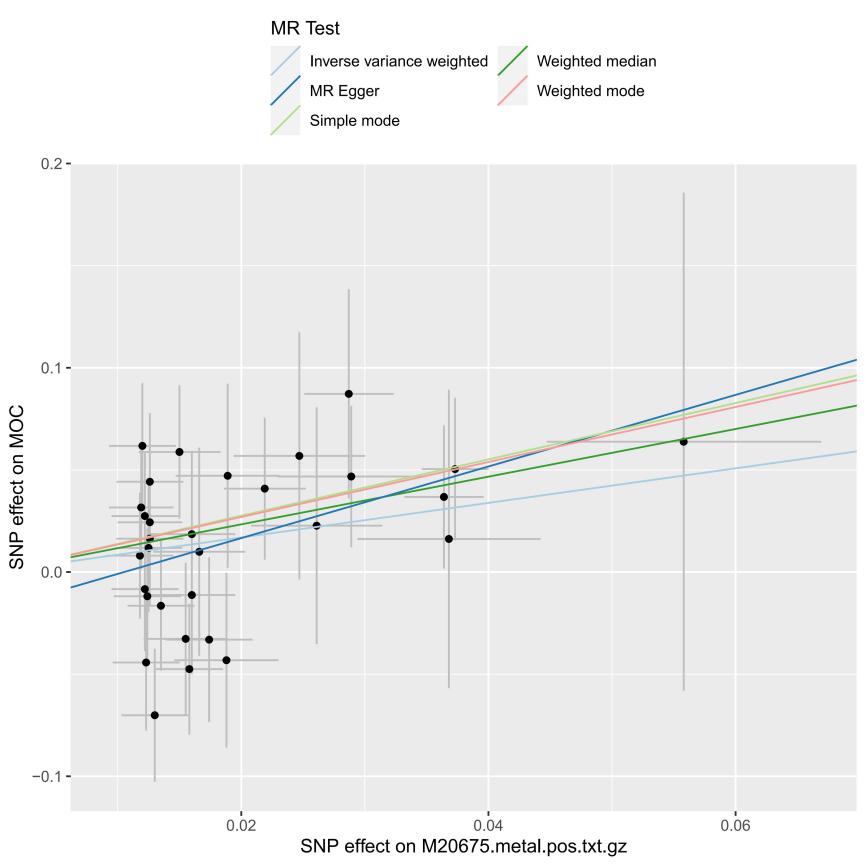

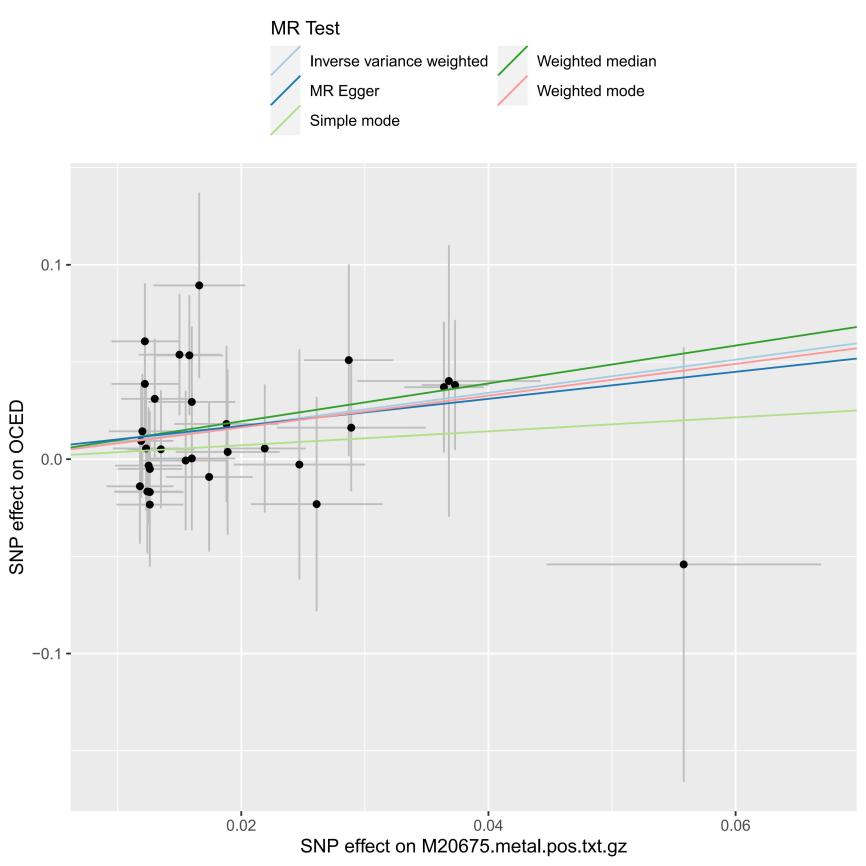


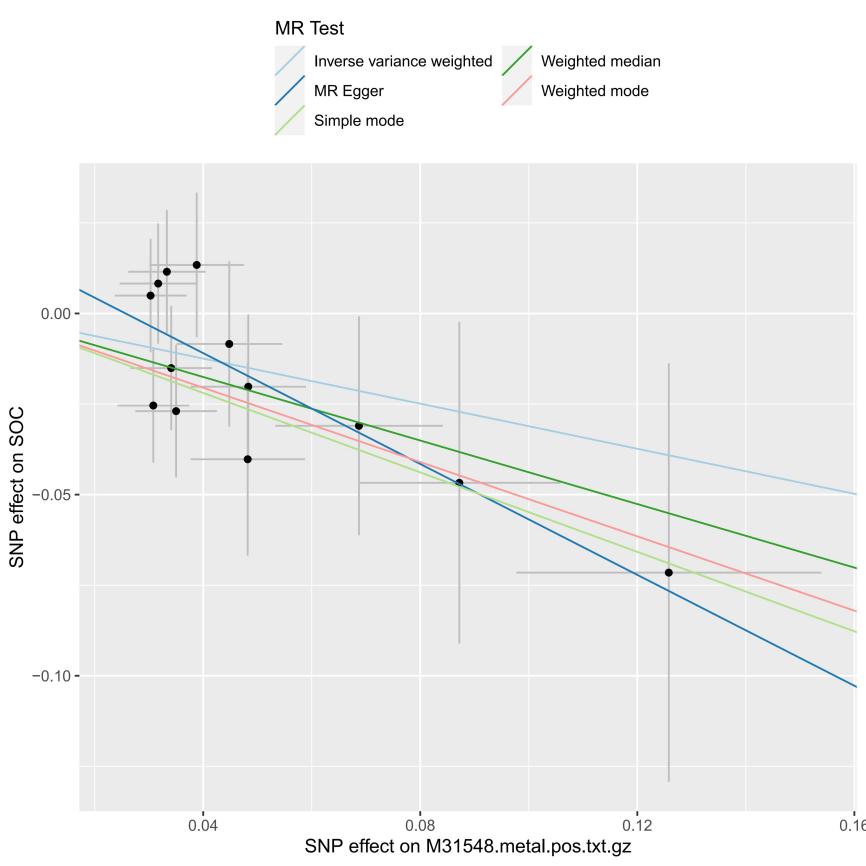

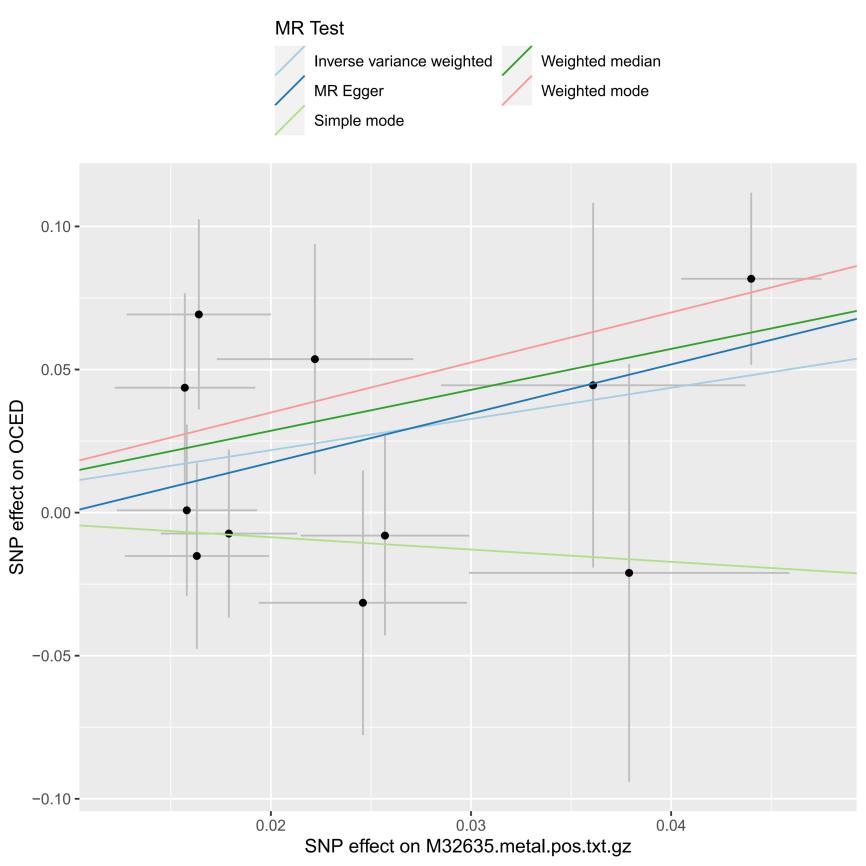


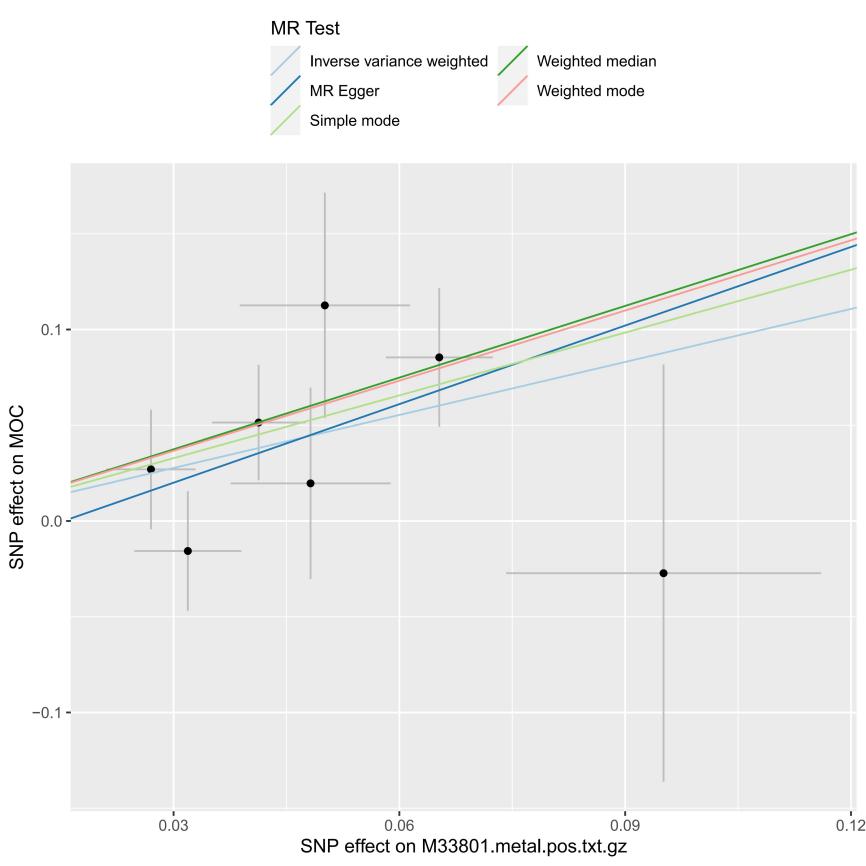

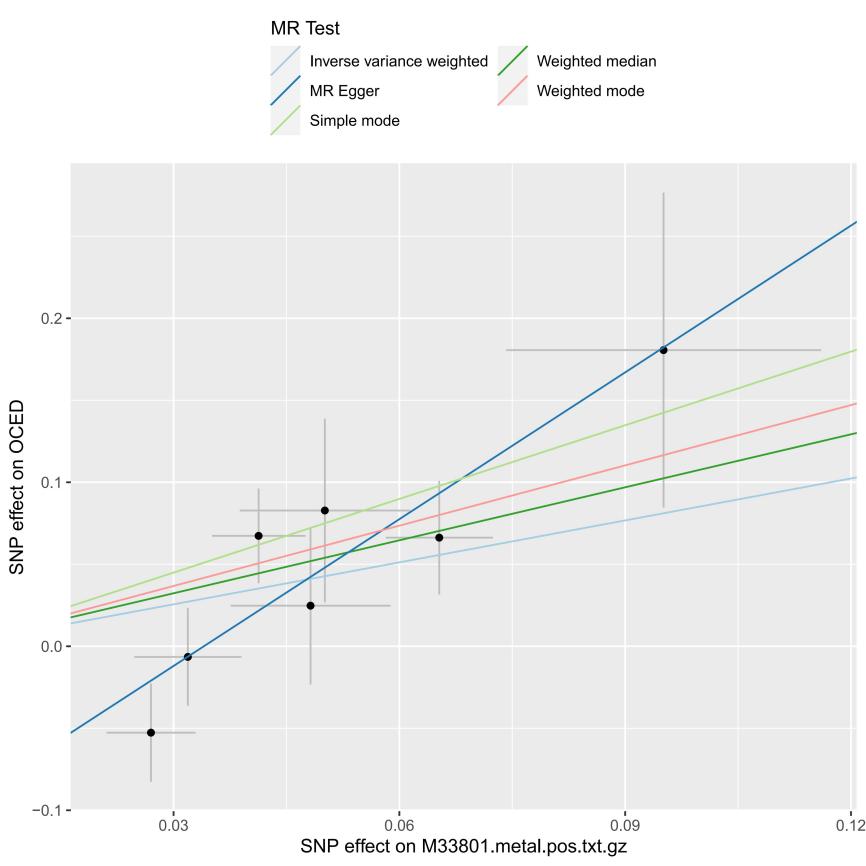


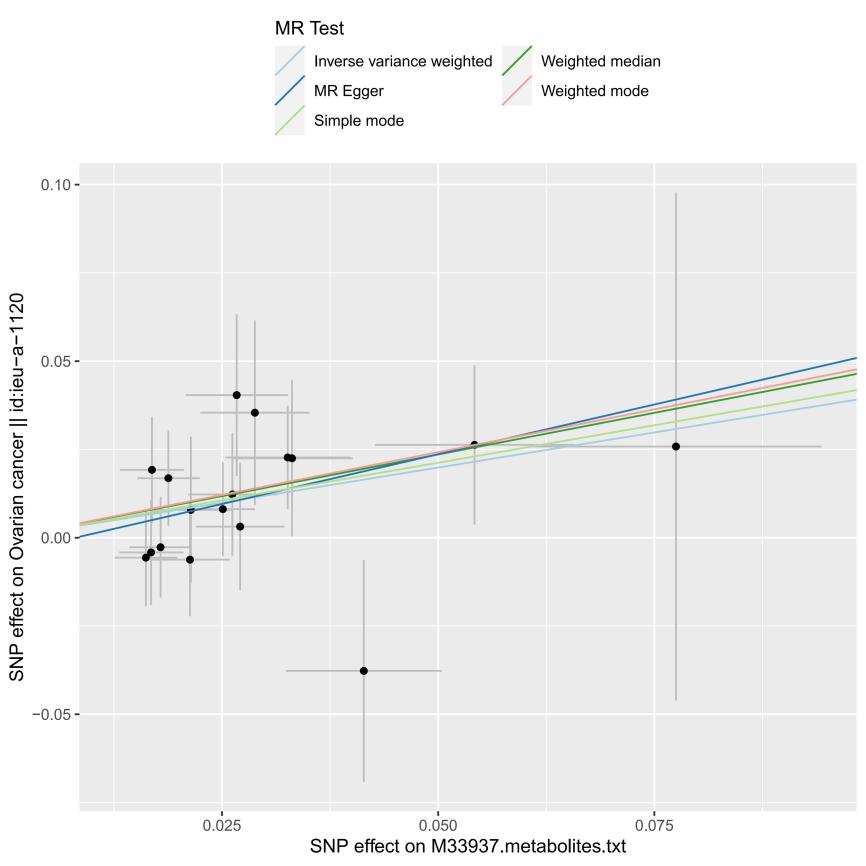

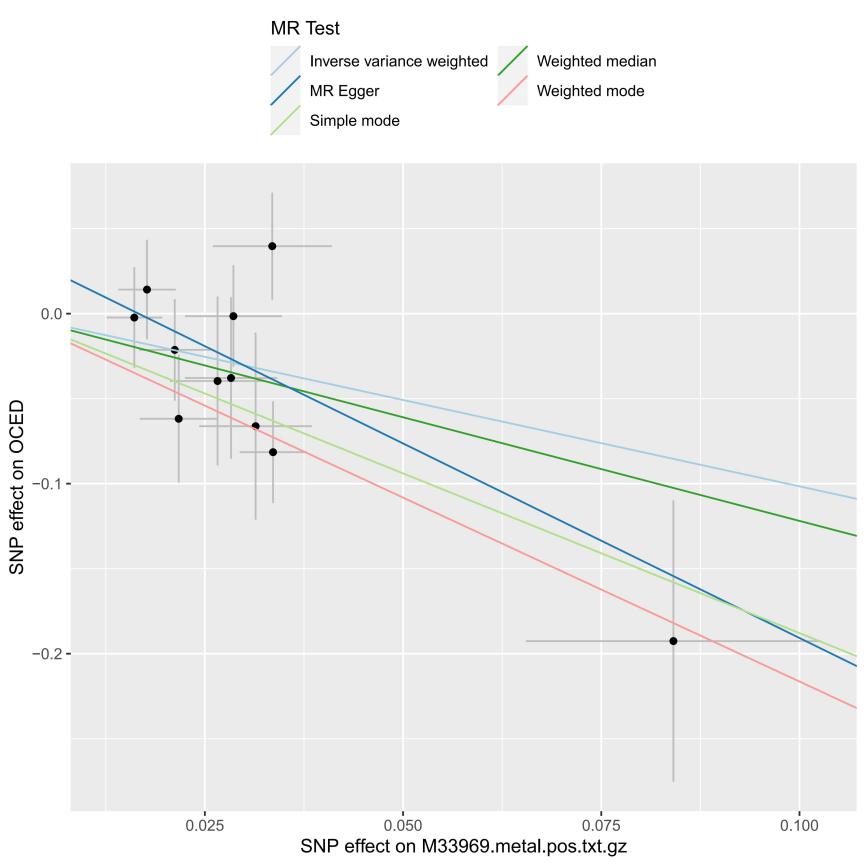


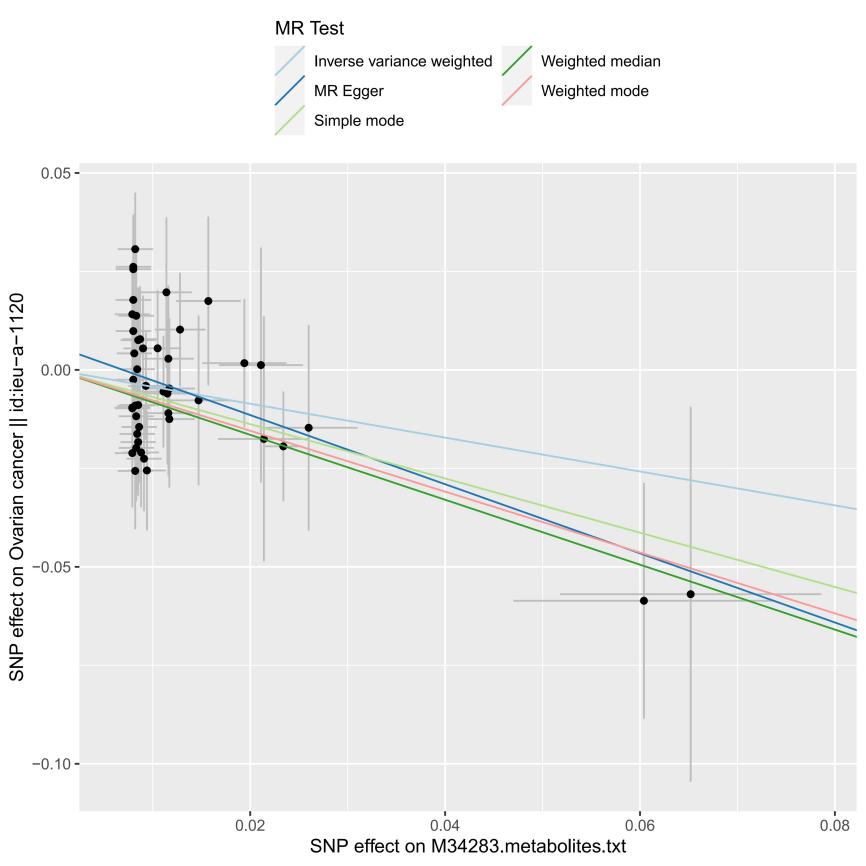

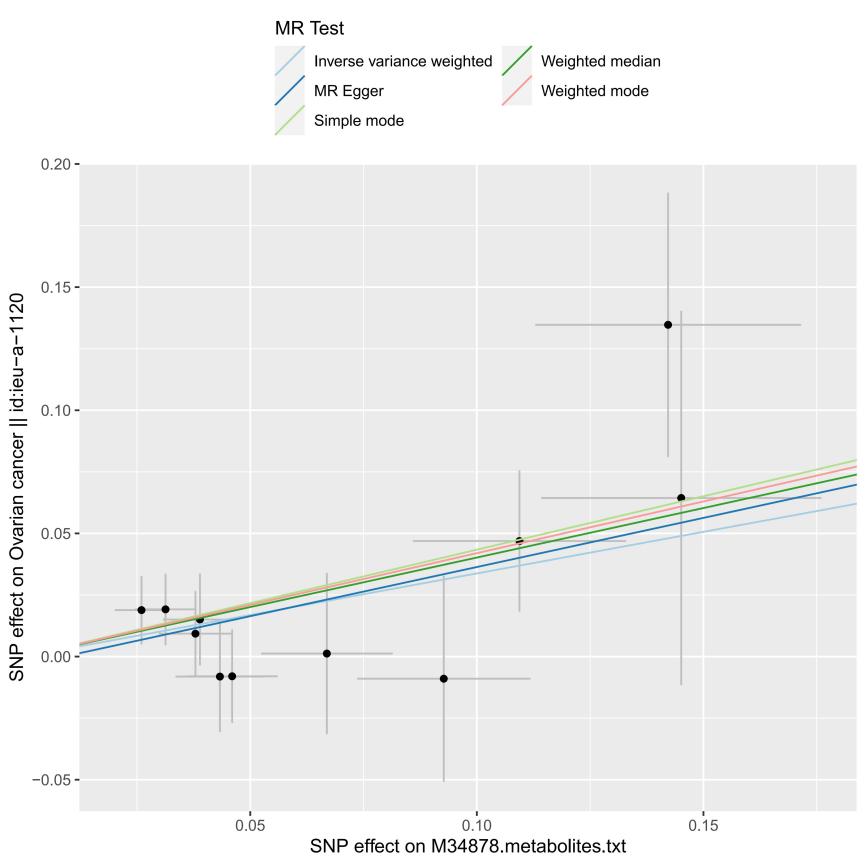


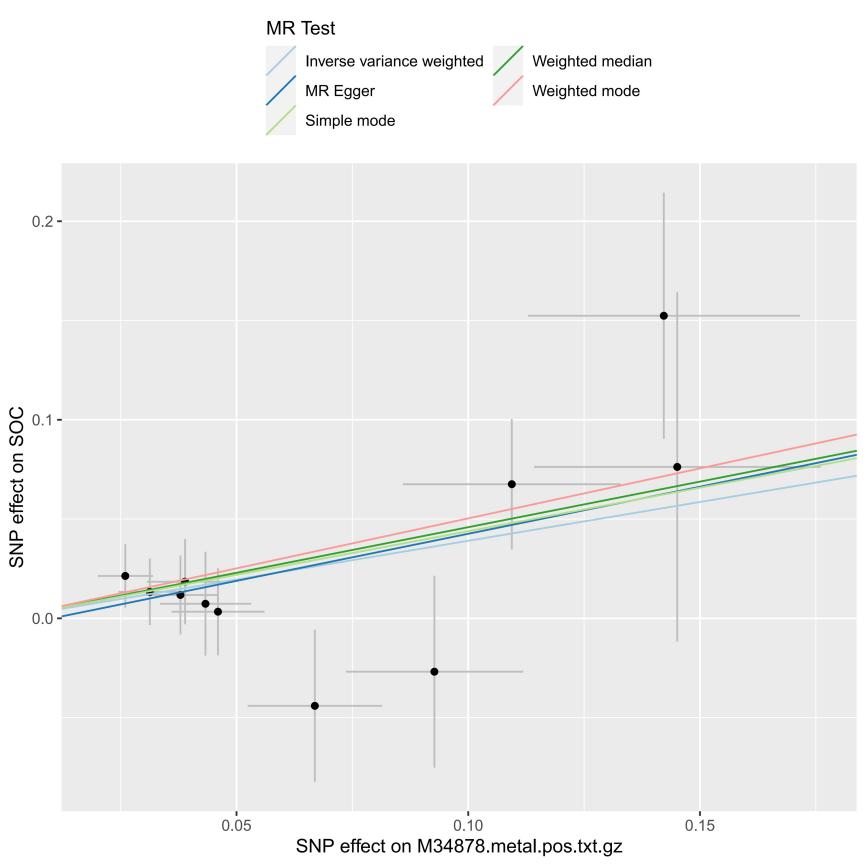

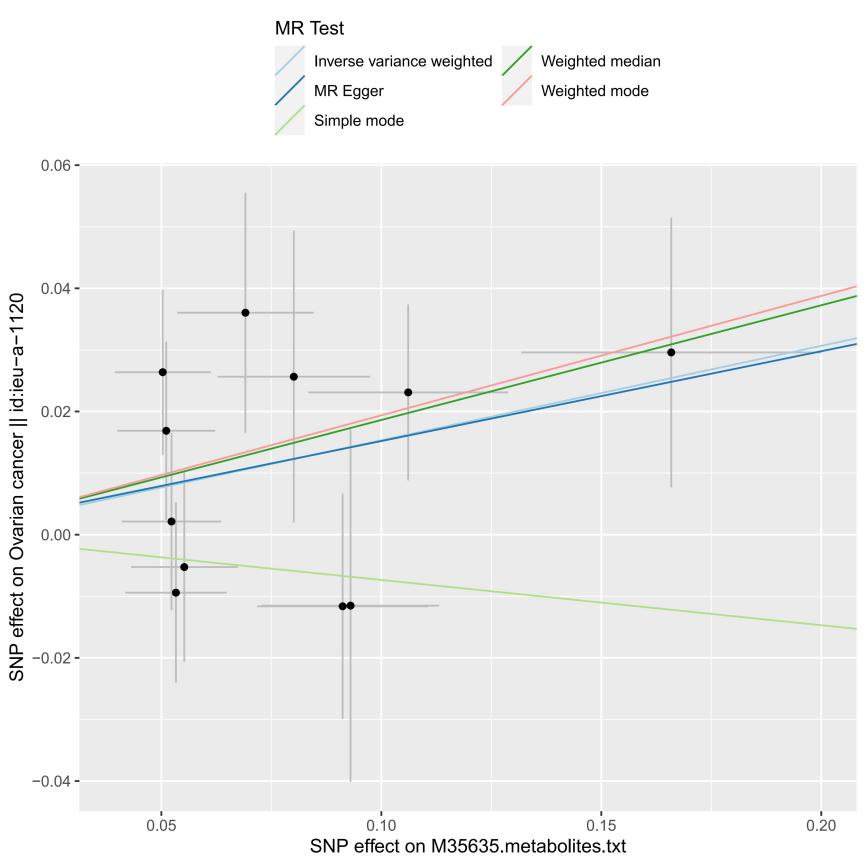


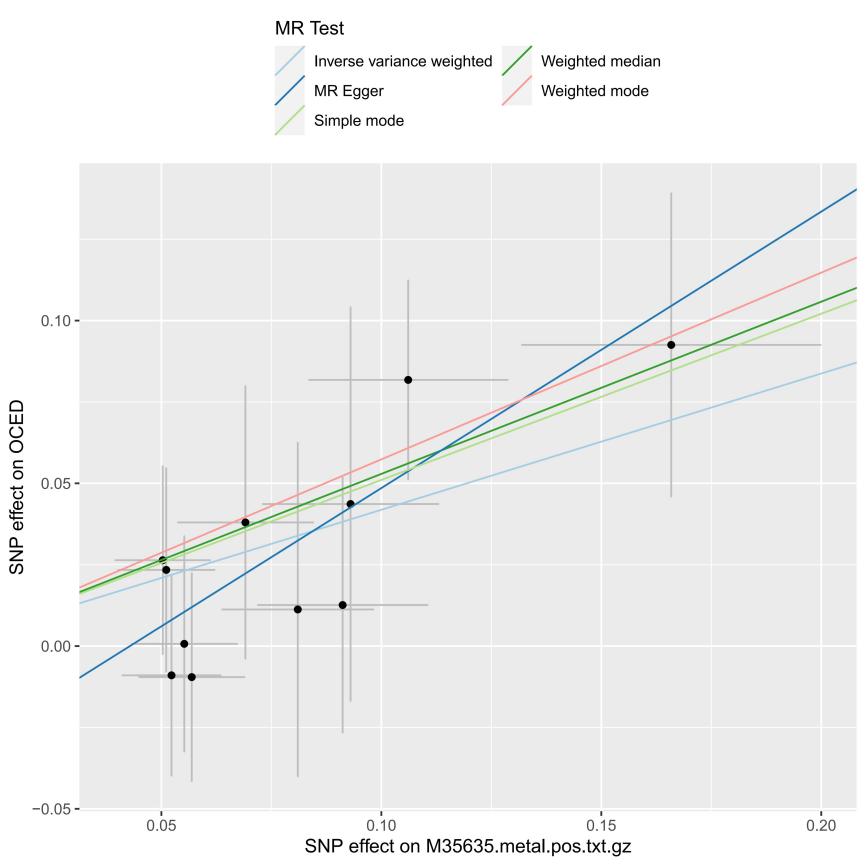

Supplement: Supplementary file 3 — Additional file 3: Supplementary Figure S2. Scatter plots of the metabolites-SNP associations (x-axis) versus the OC-SNP associations (y-axis) were shown. [file 13048_2023_1340_MOESM3_ESM.docx]
